# Low temperature traps myosin motors of mammalian muscle in a refractory state that prevents activation

**DOI:** 10.1085/jgp.201912424

**Published:** 2019-09-25

**Authors:** Marco Caremani, Elisabetta Brunello, Marco Linari, Luca Fusi, Thomas C. Irving, David Gore, Gabriella Piazzesi, Malcolm Irving, Vincenzo Lombardi, Massimo Reconditi

**Affiliations:** 1 PhysioLab, University of Florence, Florence, Italy; 2 Consorzio Nazionale Interuniversitario per le Scienze Fisiche della Materia, Firenze, Italy; 3 Randall Centre for Cell and Molecular Biophysics, King’s College London, London, UK; 4 Center for Synchrotron Radiation Research and Instrumentation and Department of Biological Sciences, Illinois Institute of Technology, Chicago, IL

## Abstract

Myosin motors in the thick filament of resting striated (skeletal and cardiac) muscle are trapped in an OFF state, in which the motors are packed in helical tracks on the filament surface, inhibiting their interactions with actin and utilization of ATP. To investigate the structural changes induced in the thick filament of mammalian skeletal muscle by changes in temperature, we collected x-ray diffraction patterns from the fast skeletal muscle extensor digitorum longus of the mouse in the temperature range from near physiological (35°C) to 10°C, in which the maximal isometric force (*T*_0_) shows a threefold decrease. In resting muscle, x-ray reflections signaling the OFF state of the thick filament indicate that cooling produces a progressive disruption of the OFF state with motors moving away from the ordered helical tracks on the surface of the thick filament. We find that the number of myosin motors in the OFF state at 10°C is half of that at 35°C. At *T*_0_, changes in the x-ray signals that report the fraction and conformation of actin-attached motors can be explained if the threefold decrease in force associated with lowering temperature is due not only to a decrease in the force-generating transition in the actin-attached motors but also to a twofold decrease in the number of such motors. Thus, lowering the temperature reduces to the same extent the fraction of motors in the OFF state at rest and the fraction of motors attached to actin at *T*_0_, suggesting that motors that leave the OFF state accumulate in a disordered refractory state that makes them unavailable for interaction with actin upon stimulation. This regulatory effect of temperature on the thick filament of mammalian skeletal muscle could represent an energetically convenient mechanism for hibernating animals.

## Abstract (precis)

The active force of mammalian skeletal muscle is reduced at low temperatures. Caremani et al. reveal that this is due to the rise of a population of myosin motors captured in a refractory state insensitive to muscle activation.

## Introduction

Contraction of striated (skeletal and cardiac) muscles is generated at the level of the sarcomere, the fundamental structural unit, by cyclical ATP-driven interactions of myosin motors extending from the thick filaments with the actin-containing thin filaments. According to the longstanding model of regulation of striated muscle, contraction is initiated by an increase in intracellular Ca^2+^ concentration, induced by membrane depolarization during the action potential, followed by Ca^2+^-dependent structural changes in the regulatory proteins of the thin filament that make the actin sites available for binding of the myosin motors (Ebashi et al., 1969; Huxley, 1973; Gordon et al., 2000). Recently, a second regulatory mechanism has emerged in which the myosin motors themselves are considered to be unavailable in resting muscle because they are trapped in helical tracks on the surface of the thick filament in which interactions with actin and ATP hydrolysis are inhibited (Woodhead et al., 2005; Stewart et al., 2010; Linari et al., 2015; Irving, 2017).

X-ray diffraction studies on demembranated fibers of mammalian skeletal muscle provided the first information concerning the temperature sensitivity of the structural and biochemical states of the myosin motors in relaxed muscle. The first myosin x-ray layer line reflection (ML1), originating from the three-stranded helical arrangement of myosin motors on the surface of the thick filament, is intense at temperatures close to physiological but becomes much weaker as temperature is lowered (Malinchik et al., 1997; Xu et al., 1997), indicating that the helical order of the motors is lost. A parallel increase in the ratio of the intensities of the 1,1 and 1,0 equatorial reflections associated with the hexagonal lattice of thick and thin filaments (*I*_1,1_/*I*_1,0_) indicates that the motors have moved away from the thick filament toward the thin filament. These changes are accompanied by a ∼1% increase in the spacing of the myosin-based meridional reflections, indicating an increase in the thick filament length (Lowy et al., 1991; Xu et al., 1997). The effect of temperature on the structure of the thick filaments of mammalian muscles was found to be related to the state of the nucleotide in the catalytic site of the myosin motor: at physiological temperature, the equilibrium favors the ATP hydrolysis products (ADP and orthophosphate [Pi]) and the motors can take up the ordered helical structure around the backbone of the thick filament; at low temperatures, the equilibrium is shifted toward the ATP state, with the motors no longer able to form the helical array and moving away from the surface of the thick filament (Schlichting and Wray, 1986; Wray, 1987; Lowy et al., 1991; Xu et al., 1997, 1999, 2002, 2003). Similar temperature-dependent changes in the intensity of the ML1 reflection and in the equatorial intensity ratio have been found in skinned trabeculae from rabbit heart in relaxing solution (Xu et al., 2006), suggesting that this mechanism is conserved throughout striated muscles.

Electron microscopy of isolated thick filaments from striated muscle has shown that under relaxing conditions, the myosin motors lie on the surface of the thick filament, folded back toward the center of the sarcomere. This conformation, known as the interacting heads motif (IHM) or OFF state, is stabilized by intramolecular interactions between the two motor domains of each myosin dimer and its motor and tail domains and intermolecular interactions between myosin molecules and other thick filament proteins (Woodhead et al., 2005; Zoghbi et al., 2008). Activation of the thick filament requires breaking these inter- and intramolecular interactions so that motors move away from the backbone of the thick filament and become available for interaction with actin (the ON state). Thick filament activation is also controlled by the level of phosphorylation of myosin binding protein C (MyBP-C, the C-terminus of which is bound to the thick filament backbone in the central one-third of the half-sarcomere [C-zone]) and of the myosin regulatory light chain (RLC; Moos et al., 1978; Herron et al., 2001; Colson et al., 2010, 2012; Kampourakis et al., 2016). In addition, in situ enzyme kinetics studies revealed a state of mammalian muscle myosin called the super relaxed state (SRX), which hydrolyses ATP at a rate 10 times slower than that observed in solution and is disrupted by interventions that destabilize the OFF state of the thick filament, including lowering the temperature or increasing the degree of RLC phosphorylation (Stewart et al., 2010; Hooijman et al., 2011). Finally, x-ray diffraction from intact fibers from frog skeletal muscle and intact trabeculae from rat heart demonstrated that thick filament activation during contraction depends on the mechanical load (Linari et al., 2015; Reconditi et al., 2017; Piazzesi et al., 2018).

In summary, the changes in the x-ray reflections that during force development in an intact muscle cell signal the loss of the ordered resting structure of the thick filament and the movement of the motors away from the filament backbone (decrease in intensity of ML1, increase in the equatorial intensity ratio [*I*_1,1_/*I*_1,0_], ∼1% increase in the extension of the thick filament) confirm the interpretation of the effects of lowering temperature or increasing the degree of MyBP-C or RLC phosphorylation in relaxed skinned preparations, in terms of switching ON the myosin motors. In structural terms, this corresponds to disruption of the IHM motif and, in biochemical terms, to loss of the SRX state.

However, to regard the IHM state seen by electron microscopy as the unique structural counterpart of the biochemical SRX state, in which ATP hydrolysis is inhibited, may be an oversimplification. Combining tryptophan fluorescence studies in solution (Málnási-Csizmadia et al., 2000, 2001) and skinned fiber x-ray diffraction (Xu et al., 1999, 2002), it was found that in the disordered prehydrolysis ATP state promoted by lowering the temperature, the ATP binding pocket is open, while in the ordered ADP.Pi state promoted by increasing the temperature, the pocket is closed. However, the strict temperature-dependent correlation between the state of the ligand in the nucleotide binding pocket and head conformation and degree of order had to be revised following the demonstration that temperature affects the head conformation and order in the same way when the ligand is substituted with a nonhydrolysable analogue of the ATP (AMP-PNP or ADP.BeF) or of the ATP-ADP.Pi transition state (ATP.Vi; Xu et al., 2003). It was concluded that motor structure is not uniquely defined by the ligand in its catalytic site, as also suggested by crystallographic studies of a motor domain in the open detached conformation in the presence of ADP (Houdusse et al., 1999).

The dependence of the structure of the thick filament of mammalian muscle on temperature in the range from near physiological (35°C) to 10°C, in which the active force is reduced by two thirds, is investigated here by recording the two-dimensional x-ray diffraction patterns at rest and at the plateau of isometric tetanic force in the intact extensor digitorum longus (EDL) muscle of the mouse. We found that lowering the temperature produces similar reductions in the fraction of myosin motors in the OFF-ordered state in resting muscle and in the fraction strongly attached to actin during active contraction, suggesting that, in contrast with the simplest hypothesis of a direct correlation between the functional OFF/ON and structural ordered/disordered populations of myosin motors, lowering the temperature promotes the rise of a population of disordered motors that are functionally OFF in the sense that they cannot bind to actin and generate active force upon stimulation. These disordered refractory motors may play an energetically convenient role in vivo in hibernating mammals.

## Materials and methods

### Muscle preparation

Male mice (*Mus musculus*, strain C57BL/6), aged 4–6 wk, were sacrificed by cervical dislocation after inhalation of anesthetic (isoflurane) according to both the Italian regulation on animal experimentation (authorization no. 956/2015 PR), in compliance with Decreto Legislativo 26/2014 and the EU regulation (directive 2010/63), and the protocols approved by the Illinois Institute of Technology Institutional Animal Care and Use Committees. The EDL muscle was dissected from the hind limbs using scissors and forceps under a stereomicroscope and mounted in a trough containing physiological solution (composition in mM: NaCl, 119; KCl, 4.7; CaCl_2_, 2.5; MgSO_4_, 1.0; NaHCO_3_, 25; KH_2_PO_4_, 1.2; and glucose, 1.1; pH 7.4 at 24°–26°C) equilibrated with carbogen (95% O_2_, 5% CO_2_). The temperature of the bathing solution was selected and kept constant (±0.2°C) by means of a servo-controlled thermoelectric module.

One tendon of the muscle was tied to a pedestal fixed to the trough and the other to the lever of a motor/force transducer system (300C-LR; Aurora Scientific) mounted on a micromanipulator for adjustment of the muscle length. Electrical stimuli were delivered by platinum plates parallel to the muscle on either side at a voltage 1.5× that required to stimulate all the fibers of the muscle. The length of the muscle was adjusted to that at which isometric tetanic force was maximum (*L*_0_).

The force was measured by interfacing the transducer system to a computer with mounted multifunctional I/O cards (PCI-6110E and PCI-DIO-32HS; National Instruments). The computer was also used for controlling the timing of stimulus, shutter operation, and synchronization with x-ray data acquisition. The program for signal generation and data acquisition was developed in house using the LabVIEW software environment (National Instruments).

### Experimental protocol

The muscle was vertically mounted on the x-ray path of the BioCAT beamline 18ID (Fischetti et al., 2004) at the Advanced Photon Source (Argonne, IL). Mounting the muscle vertically takes advantage of the smaller beam size along the meridional axis (parallel to the muscle axis), which allows for the higher resolution required to observe the fine structure of the meridional reflections (Bordas et al., 1995; Linari et al., 2000; Huxley et al., 2006b). The x-ray flux at the sample was ∼10^13^ photon ⋅ s^−1^ at 0.1-nm wavelength, with a beam size ∼350 × 350 µm (horizontal × vertical; full width at half maximum) at the sample and ∼160 × 65 µm at the detector. The beam was attenuated for muscle alignment, a fast shutter (opening time <1 ms) upstream of the sample controlled the duration of the exposure window, and the trough was oscillated in the beam during the exposure to spread the x-rays over the sample and reduce radiation damage.

X-ray patterns were collected on a high-sensitivity, high-spatial-resolution, two-chip charge-coupled device detector (Aviex PCCD1680) of active area 80 × 160 mm, with 2,084 × 4,168 pixels, each 39 × 39 µm. The phosphors in front of the detector for the x-ray to visible light conversion provided a point spread function of 65 µm. The detector was placed 3.5 m from the sample, with the longer axis parallel to the muscle axis, allowing the pattern to be collected up to ∼0.22 nm^−1^ and 0.11 nm^−1^ in reciprocal space along the meridional and equatorial (perpendicular to the meridional) axes, respectively. Two-dimensional x-ray patterns were collected at six different temperatures: 10°, 15°, 20°, 25°, 30°, and 35°C. Tetani were elicited with trains of stimuli at the frequency selected for complete fusion of the mechanical response (80, 120, 130, 130, 150, and 175 Hz). At each temperature, the muscle was exposed alternatively at rest and at the plateau of tetanic contraction. For either state, two 20-ms exposure windows were collected for a total of 40 ms per state at each temperature. The exposure windows during tetanic contraction started at different times after the onset of stimulation, according to the temperature-dependent rise time of force: from the lowest to the highest of the six temperatures, the exposure times started at 300, 180, 100, 90, 60, and 60 ms from the first stimulus.

The order of the six temperatures was randomly distributed in each muscle. Six muscles were used, with length *L*_0_ = 9.5 ± 1.6 mm and wet weight (Ww) 10.5 ± 1.2 mg (mean ± SD), from which the cross-sectional area was calculated as 2 × Ww/(ρ × *L*_0_), where ρ = 1.06 g/cm^3^ is the density of the muscle (Burkholder et al., 1994).

### X-ray data analysis

X-ray diffraction data were analyzed using FIT2D (Hammersley, 2016) and SigmaPlot (Systat Software). Two-dimensional patterns were centered and aligned using the equatorial 1,0 reflections and then quadrant folded. The distribution of diffracted intensity along the meridional axis of the x-ray pattern was obtained by integration from 0.015 nm^−1^ on either side of the meridian for the myosin-based M1, M2, …, M6 reflections and troponin-based T1 reflection. Background intensity distributions were fitted using a smoothed convex hull algorithm and subtracted. Integrated intensities of the meridional reflections were obtained from the following axial regions: M1, 0.021–0.025 nm^−1^; T1, 0.025–0.027 nm^−1^; M3, 0.068–0.072 nm^−1^ (rest), 0.066–0.070 nm^−1^ (contracting); M4, 0.093–0.095 nm^−1^; M5, 0.115–0.118 nm^−1^; and M6, 0.137–0.142 nm^−1^ (rest), 0.135–0.140 nm^−1^ (contracting), and their center of mass, i.e., the spacing of the reflection, was determined. The intensity of the M2 reflection was extracted by fitting multiple Gaussian peaks to the cluster between 0.046 and 0.049 nm^−1^, considering the “true” M2 reflection only the peak at spacing ∼0.0464 nm^−1^, which indexes on the axial 43-nm periodicity on the thick filament. The interference components of the M3 reflection in the contracting muscle were determined by fitting multiple Gaussian peaks, with the same axial width, to the meridional intensity distribution, and the total intensity of the reflection was calculated as the sum of the component peaks, giving the same result as the total integrated intensity within the limits reported above. The radial, cross-meridional width of the M3 and M6 meridional reflections was determined from Gaussian fits of the integrated radial intensity distribution in the axial regions defined above for each reflection. For both M3 and M6, the radial width at rest was the same independent of temperature, while during isometric contraction the radial width of M3 increased by ∼1.5 at 10°C and 2 at 35°C, relative to the values at rest, and that of M6 increased by ∼2.2 independent of temperature. The increase of the radial width in isometric contraction is a consequence of lateral misalignment between ﬁ laments induced by the rise in force. This misalignment produces by itself an intensity decrease that is corrected by multiplying the observed intensity by the cross-meridional width (Huxley et al., 1982).

The equatorial intensity distribution was determined by integrating between 0.0036 nm^−1^ on either side of the equatorial axis, and the intensities and positions of the 1,0 and 1,1 reflections were determined by a Gaussian fit. At rest, the so-called Z reflection, which arises from the square lattice of the thin filaments where they are anchored to the Z-line, partially overlaps with the 1,1 reflection, and the Gaussian fit allows them to be separated. During contraction, due to the widening of the 1,1 reflection, it was not possible to extract the contribution of the Z line to the 1,1 reflection, so its effect was neglected.

The intensity profile of the myosin- and actin-based layer lines in the direction parallel to the meridional axis was obtained by integrating between 0.018 and 0.076 nm^−1^ from the meridional axis. At rest, it was not possible to separate the contribution of the first-order actin layer line (AL1) that is centered at ∼0.0265 nm^−1^ and overlaps with the high-angle (HA) side of the first-order myosin layer line (ML1). Therefore, the temperature dependence of the ML1 intensity at rest was determined by integrating the intensity of its low-angle (LA) side (0.019–0.024 nm^−1^; Piazzesi et al., 1999). In isometrically contracting muscle, the contributions of ML1 (or the corresponding actomyosin reflection, see Discussion) and AL1 have been separated by a two-Gaussian fit, with the constraint that their axial width is the same. The radial profiles of the ML1 at rest were obtained by integrating between 0.019 and 0.024 nm^−1^ along the meridional direction, as described above, and the underlying background was estimated by applying the smoothed convex hull algorithm to slices 50 pixels (or 0.0057 nm^−1^) wide along the meridional axis and subtracted.

The spatial calibration of the patterns was obtained by collecting with the same experimental setup a diffraction pattern from a small bundle of fibers from the frog muscle (tibialis anterior, 4°C) and from the mouse EDL muscle (30°C) at the ID02 beamline at the European Synchrotron Radiation Facility (ESRF; Grenoble, France; Narayanan et al., 2018), which provided ≤2 × 10^13^ photon ⋅ s^−1^ with 0.1-nm wavelength in a beam of size ∼300 × ∼50 µm (horizontal × vertical, full width at half maximum) at the sample. The position of the strong M3 reflection, for which a spacing of 14.34 nm is assumed (Haselgrove, 1975), was the same in the two preparations, within experimental error (∼0.01%).

The amount of sarcomere length shortening during tetanic force development and the force–sarcomere length relation in our EDL muscle preparation was determined by vertically mounting a muscle, prepared as described above, at the ID02 beamline (temperature 29°C). The ultra-small-angle reflections originating from the sarcomere repeat were collected with 2-ms exposure on a charge-coupled device–based fast readout/low noise (FReLoN) detector placed 31 m from the sample, from the muscle at different lengths, both at rest and at the plateau of the isometric tetanus.

Six animals were used for the experiments: five at Advanced Photon Source (10 muscles) for the temperature experiments and one at ESRF for the sarcomere length test (2 muscles). Data reported for temperature experiments refer only to the six muscles for which the temperature series was complete.

### Online supplemental material

Fig. S1 shows temperature-dependent intensity profiles and spacing of the equatorial reflections, both at rest and at the plateau of the isometric tetanus. Fig. S2 shows temperature-dependent intensity profiles of the meridional reflections. Fig. S3 shows the temperature-dependent intensity profile of the ML1 layer line along the radial direction. Fig. S4 shows the theoretical relation between intensity of the AL1 layer line and the fraction of attached motors. Fig. S5 shows the temperature-dependence of the fraction of actin-attached motors determined from the intensity of AL1 for different assumptions on the attached fraction during isometric contraction at 30°C. Supplemental text describes the fraction of motors attached to actin in isometric contraction and estimates the fraction of attached motors from the intensity of the first actin-based layer line.

## Results

### Temperature dependence of isometric force production

**Figure 1.**
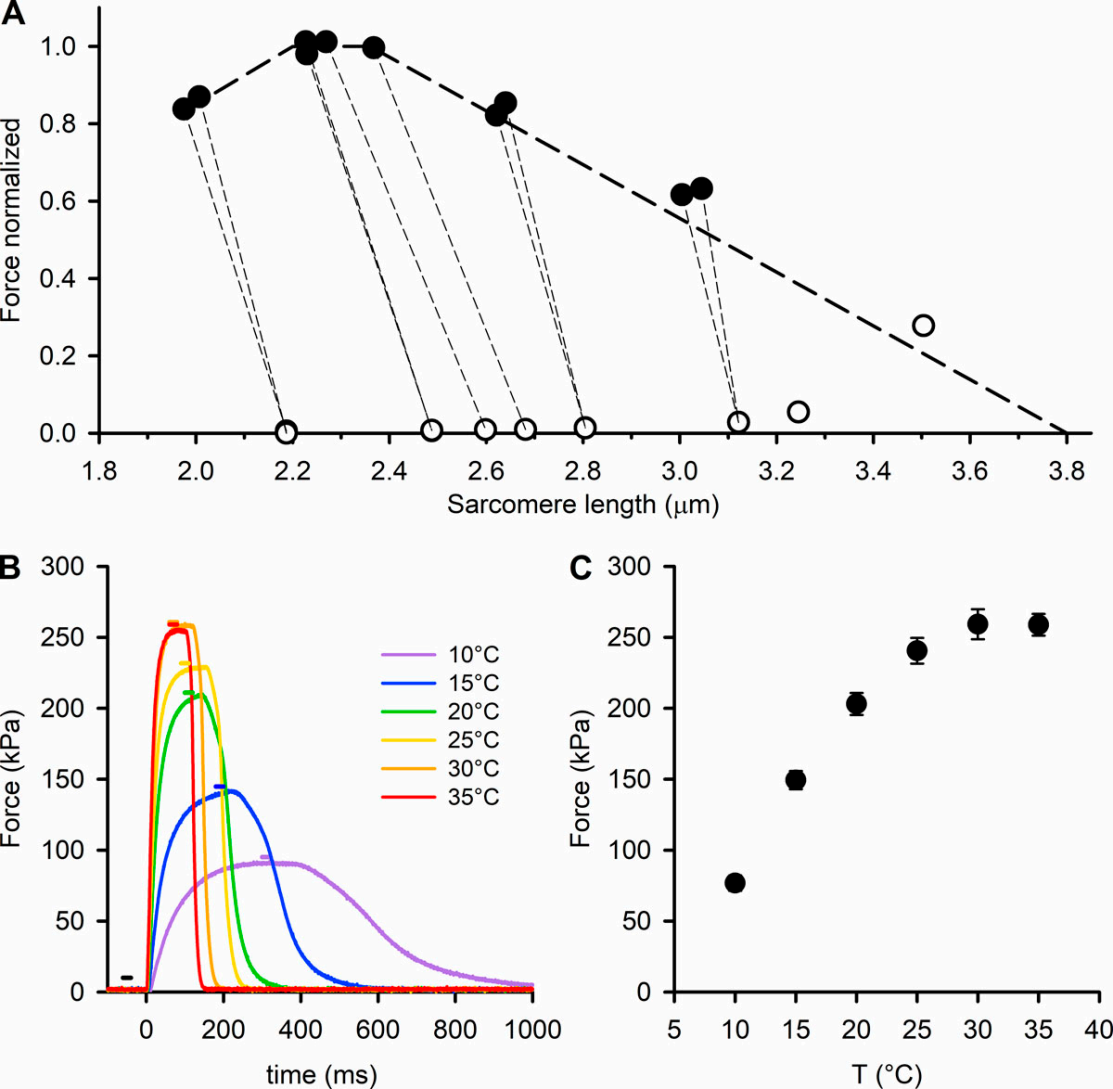
**Temperature dependence of the isometric force developed by the EDL muscle. (A)** Relation between sarcomere length (SL) and force either at rest (open circles) or at the isometric tetanus plateau (filled circles). Temperature 29°C. The thin dashed lines join the rest and tetanus plateau values of the same record to show the SL shortening during force development. Measurements done at the ID02 beamline of the ESRF. The thick dashed line shows the force–SL relation calculated using the value of 2.2 µm for the total length of the thin filaments reported for mouse muscle (Close, 1972) and 0.16 µm for the bare zone (Linari et al., 2000). **(B)** Force responses to tetanic stimulation of an EDL muscle at different temperatures (color code in the inset). Time zero marks the start of the stimulation. The horizontal bars indicate the 20-ms exposure windows, with the same color code as the force traces (black for exposure at rest). **(C)** Temperature (T) dependence of the maximal isometric force (mean ± SEM, *n* = 12; six muscles).

Isometric force was measured at the muscle length (*L*_0_) at which active force was maximum, corresponding to the sarcomere length at which the actin-containing thin filaments fully overlap with the region of the thick filaments containing myosin motor domains. Because sarcomere lengths are difficult to determine by conventional optical diffraction methods in intact EDL muscles from the mouse, we used ultra-small-angle x-ray scattering for direct measurements of the resting sarcomere length and the sarcomere length attained at plateau force in fixed-end tetanic contractions. The results showed that in our preparation, *L*_0_ corresponds to a sarcomere length of ∼2.6 µm in the resting muscle and ∼2.3 µm at the tetanus plateau. Thus, during tetanic force development, a shortening of ∼10% occurs against the compliance of tendon attachments (Fig. 1 A). Tetanic force is at a maximum at sarcomere length 2.3 µm, in agreement with the force–sarcomere length relation expected (thick dashed line in Fig. 1 A) on the basis of the lengths of the filaments in mouse muscle (Close, 1972).

Force production during tetanic stimulation at fixed length *L*_0_ increased by a factor of 3 with increasing temperature in the range 10°–30°C, and the rates of force development and relaxation were higher (Fig. 1 B). Stimulus duration and frequency were adjusted to give a fused tetanus with a well-defined force plateau at each temperature. Tetanic plateau force increased steeply with temperature between 10° and 20°C, then less steeply at higher temperatures (Fig. 1 C).

### Low-angle x-ray diffraction from resting and contracting muscle

**Figure 2.**
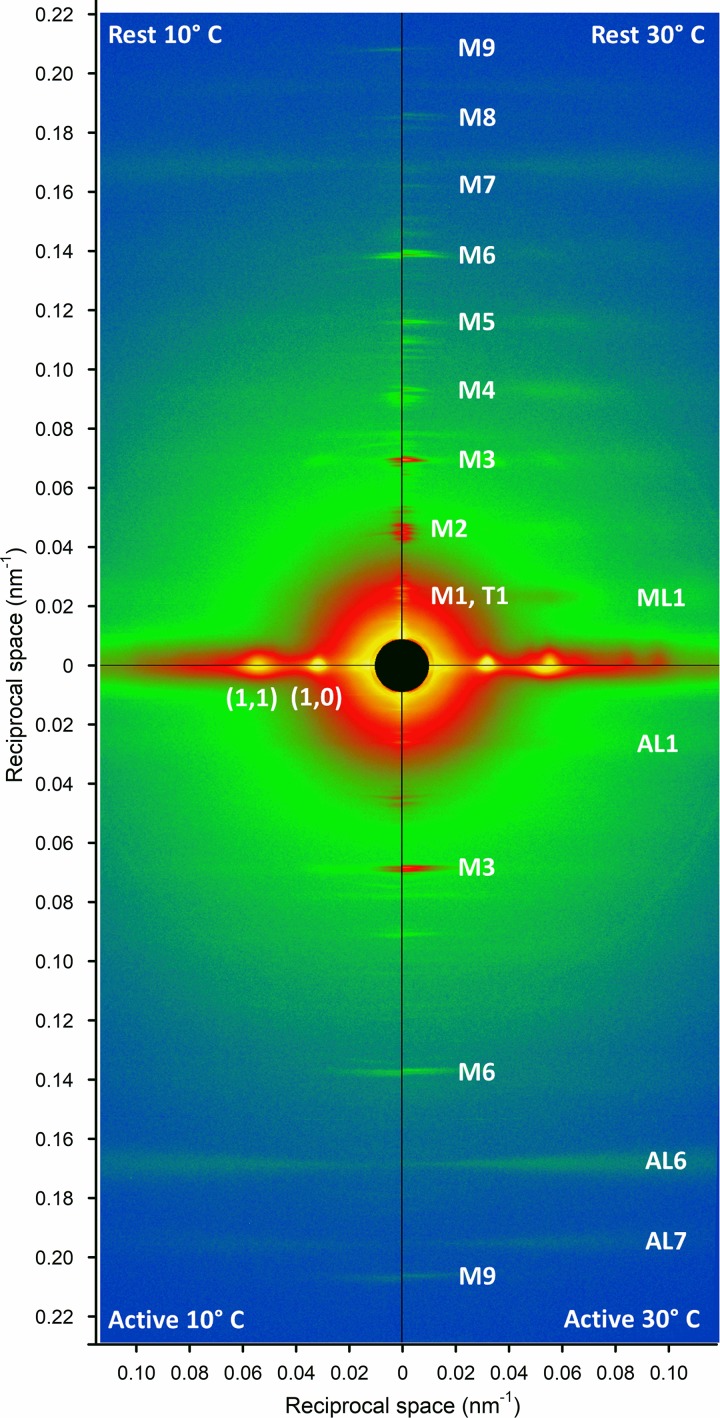
**Low-angle x-ray diffraction patterns from EDL muscle.** The left and the right quadrants are collected at 10°C and 30°C respectively, both at rest (upper quadrants) and at the plateau of the isometric tetanus (lower quadrants). The vertical (meridional) axis is parallel to the muscle axis. Each quadrant is obtained with 2 × 20-ms exposure windows with 3.5-m camera length. On the meridional axis are indicated the myosin-based (M) and troponin-based (T) reflections. On the horizontal (equatorial) axis are indicated the strong 1,0 and 1,1 reflections arising from the filament lattice. The myosin (ML) and actin (AL) layer lines that extend in the radial direction are due to the helical arrangement of the two proteins in the thick and thin filaments.

Changes in the structure of the thick and thin filaments associated with muscle activation were measured at the BioCAT x-ray beamline (18ID) of the Advanced Photon Source. At each temperature, x-ray diffraction patterns were recorded in the resting muscle and at the peak of the tetanus (horizontal bars in Fig. 1 B). These patterns (Fig. 2) show the characteristic equatorial reflections 1,0 and 1,1 associated with the hexagonal lattice of thick and thin filaments, the meridional reflections associated with the axial periodicity of the thick filaments (M1–M9), and the myosin-based and actin-based layer line reflections associated with helical periodicities in the thick and thin filaments (ML1 and AL1–AL7, respectively). The effects of muscle activation and temperature on each type of reflection are described in the following sections.

### Equatorial reflections

**Figure 3.**
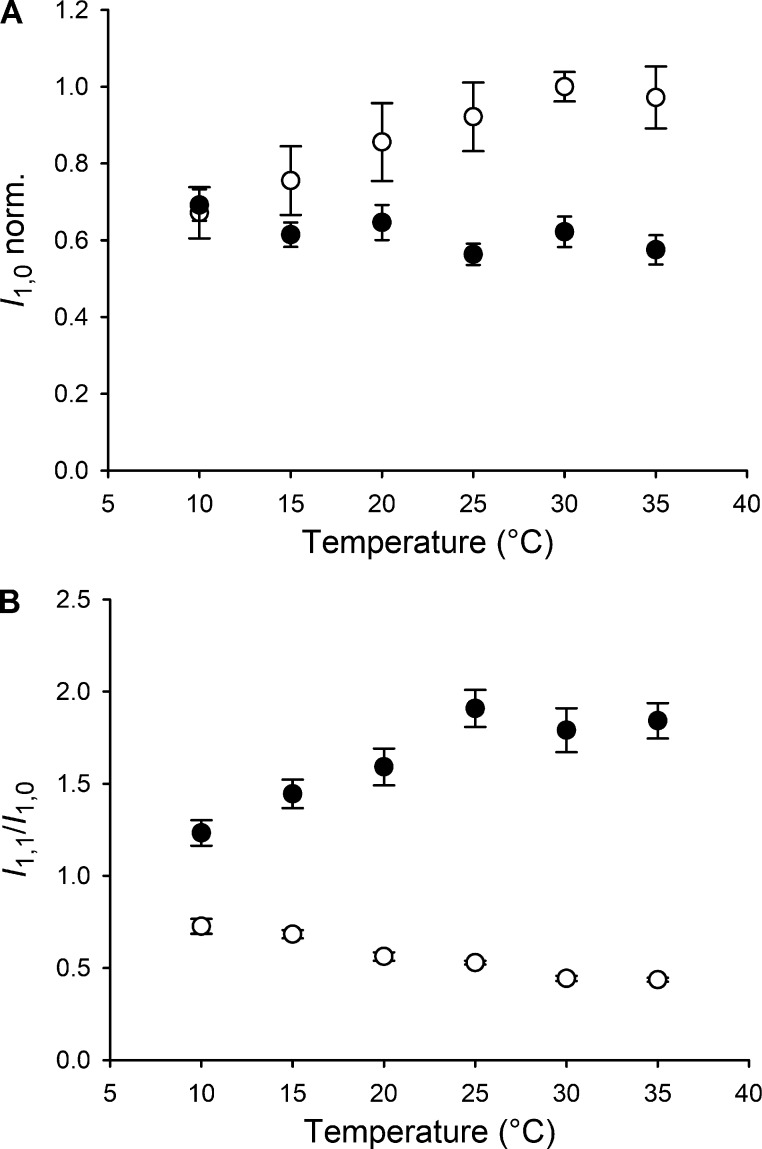
**Temperature dependence of the equatorial reflections. (A)** Intensity of the 1,0 equatorial reflection both at rest (open circles) and at the plateau of the isometric tetanus (black circles), normalized (norm.) for the value at rest at 30°C. **(B)** Intensity ratio of the 1,1 and 1,0 reflections, at rest (open circles) and at the plateau of the isometric tetanus (black circles); mean ± SEM, *n* = 12; six muscles.

In resting muscle, the equatorial 1,0 reflection, associated with the lattice planes containing the thick filaments, is relatively strong (Fig. S1 A), signaling the proximity of the myosin motors to the thick filament backbone (Haselgrove and Huxley, 1973). The intensity of the 1,0 reflection (*I*_1,0_) is greater at higher temperature in resting muscle (Fig. 3 A, open circles), although this effect is more pronounced in the lower part of the temperature range, with relatively little change above 25°C. The lower *I*_1,0_ in resting muscle at lower temperatures indicates that myosin motors move away from the thick filament surface. Contraction at higher temperature produces a larger decrease in *I*_1,0_ (Fig. 3 A, filled circles) as more myosin motors leave the thick filament surface, attach to the thin filaments, and generate force. The effect of muscle activation on *I*_1,0_ is abolished at 10°C, indicating that myosin motors have already left the thick filament surface in the resting muscle at this temperature, and there is no additional effect of activation.

The equatorial 1,1 reflection, associated with the lattice planes containing both thin and thick filaments, is relatively weak in resting muscle and becomes stronger during contraction (Fig. S1 A). The ratio of the intensities of the 1,1 and 1,0 reflections (*I*_1,1_/*I*_1,0_; Fig. 3 B) is a qualitative indicator of the fraction of motors that have left the surface of the thick filaments and become associated with the thin filaments. In resting muscle, *I*_1,1_/*I*_1,0_ decreases slightly with increasing temperature (Fig. 3 B, open circles), presumably because the myosin motors become closer to the thick filament surface. In active muscle, *I*_1,1_/*I*_1,0_ increases with temperature in the range of 10–25°C (Fig. 3 B, filled circles), with no further increase at higher temperatures, approximately following the temperature dependence of both active force (Fig. 1 C) and *I*_1,0_ in resting muscle (Fig. 3 A, open circles). The interfilament spacing, conventionally measured as the separation of the 1,0 lattice planes (*d*_10_; Fig. S1 C) decreases slightly during muscle contraction, indicating a radial component of active force (Cecchi et al., 1990; Brenner and Yu, 1991; Williams et al., 2010). The lattice compression observed in the present experiments is significantly smaller than that during isometric force development in intact fibers from frog muscle (∼4%; Cecchi et al., 1990). The difference is likely to be a consequence of the lattice expansion due to the ∼10% shortening accompanying force development in our preparation. The interfilament spacing progressively increases on cooling below 25°C at rest, suggesting that release of the myosin motors from the thick filament surface at low temperature in the absence of active force increases interfilament separation by an electrostatic or steric effect.

### Meridional reflections

The intensity distribution along the meridian of the diffraction pattern in resting muscle (Figs. S2 A and 2) consists of a series of reflections designated M and associated with orders of the fundamental myosin-based ∼43-nm axial periodicity of the thick filaments, and a T series associated with the ∼38-nm axial periodicity of troponin in the thin filaments (Huxley and Brown, 1967). During contraction, the M3, M6, and M9 reflections and the T series reflections remain strong, whereas the other myosin-based meridional reflections, the so-called forbidden reflections (M1, M2, M4, and M5; Fig. S2) associated with the perturbation of the axial repeat of three consecutive crowns within each 43-nm repeat in the C zone (Malinchik and Lednev, 1992; Reconditi et al., 2014), become much weaker. Here we focus on the well-characterized M3 and M6 reflections that remain strong during contraction and signal structural changes in the myosin motors and the thick filament backbone, respectively (Huxley et al., 1983, 2006b; Reconditi et al., 2004; Linari et al., 2015).

**Figure 4.**
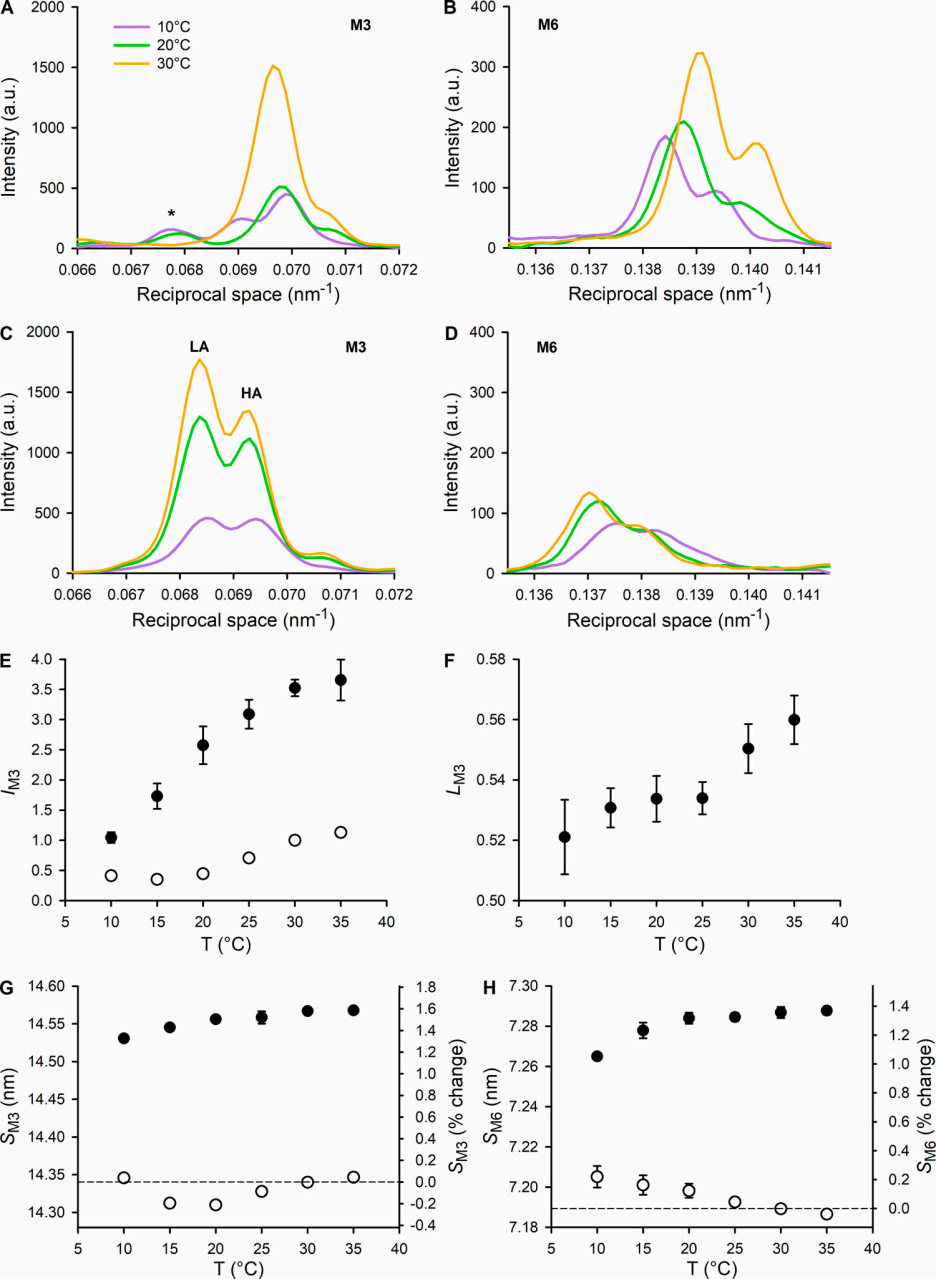
**Temperature dependence of the myosin-based meridional reflections that signal the regulatory state of the thick filament. (A and B)** Intensity profiles at rest of the M3 and M6 reflections, at 10°, 20°, and 30°C (color code in the inset). *, A peak present at the lower temperature that is not part of the M3. **(C and D)** Intensity profiles at the plateau of the isometric tetanus of the M3 and M6 reflections, at 10°, 20°, and 30°C (same color code as in A and B). The two main peaks of M3 fine structure are indicated. All the profiles shown in A–D are obtained from one EDL muscle (the same as in Fig. 2) by adding 2 × 20-ms exposures for each state and each temperature. **(E)** Temperature (T) dependence of the intensity of the M3 reflection at rest (open circles) and at the plateau of the isometric tetanus (black circles), normalized for the value at rest at 30°C. **(F)** Temperature dependence of the ratio between the intensity of the LA peak and the total intensity of the M3 (*L*_M3_) at the plateau of the isometric tetanus. **(G and H)** Temperature dependence of the spacing of the M3 and M6 reflections, respectively, at rest (open circles) and at the plateau of the isometric tetanus (black circles). Vertical scale: on the left, nm; on the right, percentage difference from the value at rest at 30°C. Data in E–H are mean ± SEM; six muscles.

The M3 reflection in resting muscle at high temperature (Fig. 4 A) has a strong peak corresponding to an axial periodicity of 14.34 nm (or 0.0697 nm^−1^ in reciprocal space) and a characteristic shoulder ∼14.14 nm (or 0.0707 nm^−1^) arising from x-ray interference between the two arrays of myosin motors in each thick filament (Linari et al., 2000). In active contraction, the M3 reflection moves to a lower reciprocal spacing (Fig. 4 C), corresponding to an ∼1.4% increase in the real-space periodicity of the myosin motors and is split into two interference subpeaks of roughly equal intensity referred to as LA and HA peaks. The M3 reflection is weaker at lower temperature in both resting (Fig. 4 A) and active (Fig. 4 C) muscle. In resting muscle, the intensity decrease on cooling is accompanied, at temperatures <25°C, by a change in the interference modulation of the reflection and by the appearance of a new peak (* in Fig. 4 A) corresponding to an axial periodicity of ∼14.80 nm (or 0.0676 nm^−1^), as reported previously in skinned fibers from rabbit psoas muscle (Xu et al., 2006). Notably, the spacing of this new peak is too large to be associated with interference between the two arrays of myosin motors in each thick filament and is therefore assigned to an unidentified structural periodicity in resting muscle at low temperature. At 10°C, the fine structure of the M3 reflection at rest changes drastically, showing an LA peak at 14.48 nm (0.00691 nm^−1^) with an intensity about half that of the main peak. Cooling has a less obvious effect on the profile of the M3 reflection during active contraction (Fig. 4 C), but there is a systematic change in the fractional intensity of the LA peak, *L*_M3_ (Fig. 4 F), which increases slightly but reproducibly with temperature increase (Linari et al., 2005). *L*_M3_ is a sensitive measure of the axial motion of the myosin motors (Reconditi, 2006; Brunello et al., 2009), and the lower *L*_M3_ at lower temperature indicates that the motors have moved axially slightly away from the midpoint of the thick filament (toward the beginning of the working stroke). The measured intensity of the M3 reflection is affected by the degree of axial alignment between adjacent thick filaments, and a first-order correction for changes in this parameter can be obtained by multiplying the measured intensity by the radial width of the reflection (Huxley et al., 1982). These corrected *I*_M3_ values (Fig. 4 E) reveal a much larger increase in *I*_M3_ during muscle contraction than previously reported in amphibian muscle (Linari et al., 2005; Reconditi et al., 2011). The dependence of *I*_M3_ on temperature in active muscle is similar to that of active force (Fig. 1 C).

The M6 reflection, which is more specifically associated with the axial periodicity of the backbone of the thick filament than the M3 reflection, is relatively insensitive to the conformation of the myosin motors. The intensity of the M6 reflection (*I*_M6_) slightly decreases on cooling in either resting (Fig. 4 B) or active contraction (Fig. 4 D) conditions, but is much less sensitive to temperature than *I*_M3_. The interference fine structure of the M6 reflection is also relatively independent of temperature in both conditions (Fig. 4, B and D). The spacing of the M6 reflection (*S*_M6_) increases by ∼1.4% during contraction at high temperature (Fig. 4 H), similar to the change in the spacing of the M3 reflection (Fig. 4 G). These increases in spacing correlate with switching ON of the thick filament (Linari et al., 2015). Cooling the resting muscle produces a smaller but reproducible increase in *S*_M6_ (Fig. 4 H, open circles) but not in *S*_M3_ (Fig. 4 G, open circles). The effect, which is not accompanied by any temperature-dependent change in resting tension, is consistent with the idea that cooling resting mammalian muscle disrupts the quasihelically ordered (OFF) state of the thick filaments (Malinchik et al., 1997; Xu et al., 1997), in the absence of active force. In contrast, *S*_M6_ during active contraction is slightly smaller at lower temperature, which is likely to be related to the lower active force (Fig. 1 C; Linari et al., 2015).

### Layer line reflections

**Figure 5.**
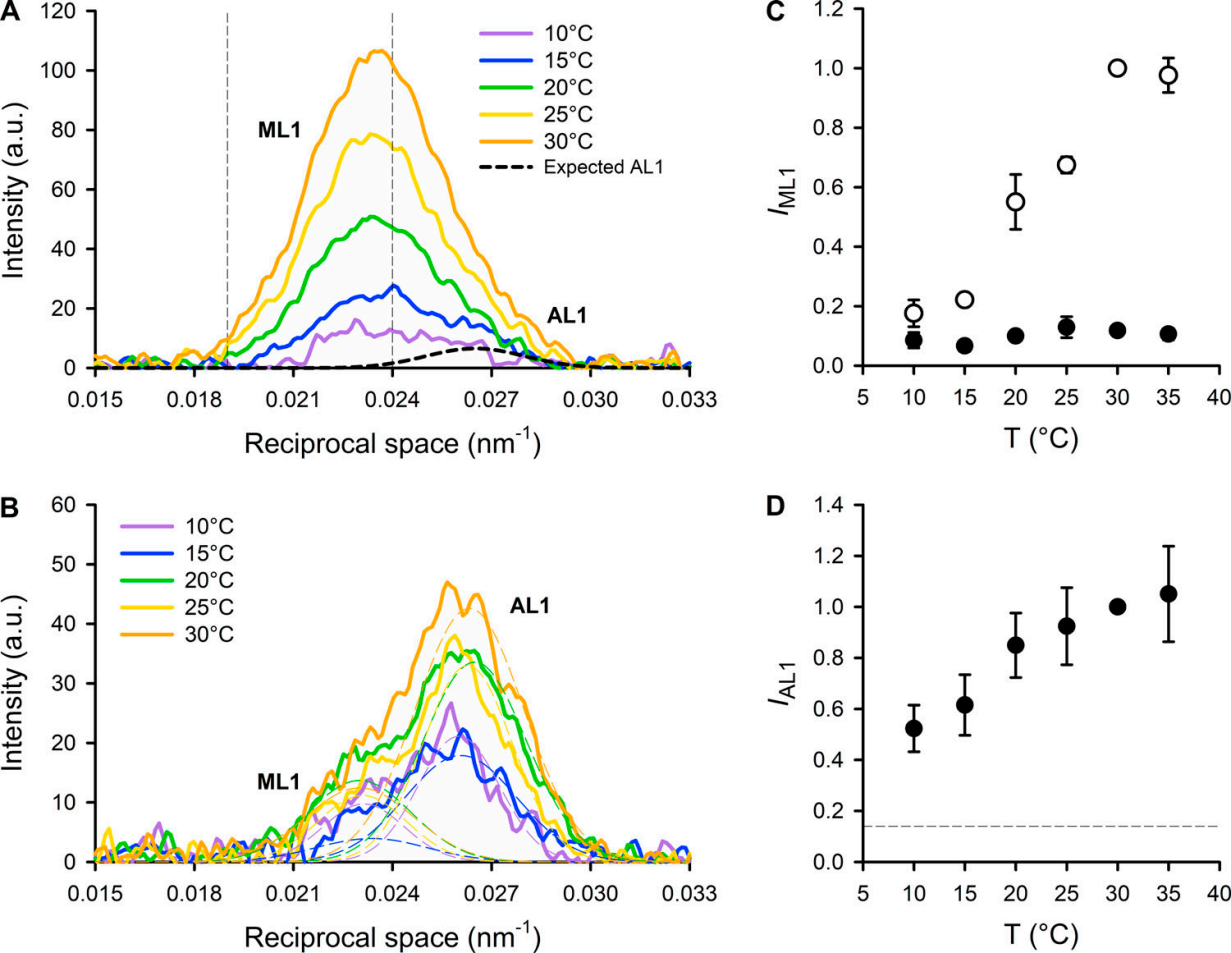
**Temperature dependence of the first myosin- and actin-based layer lines. (A and B)** Intensity profiles, parallel to the meridional axis, of the myosin- and actin-layer lines (ML1 and AL1), in the range 10°–30°C (color code in the insets) at rest (A) and at the plateau of the isometric tetanus (B), from one muscle. In A, the thin vertical dashed lines represent the limits of integration for the estimate of the temperature dependence of the intensity of ML1 (*I*_ML1_). The thick black dashed line represents the Gaussian intensity distribution expected for AL1 at rest (see text). In B, the thin dashed lines represent the results of the Gaussian fit used to separate the contribution of ML1 (left) and AL1 (right). See text for details. All the profiles shown in A and B are obtained from the same EDL muscle as in Fig. 2 by adding 2 × 20-ms exposures for each state and each temperature. a.u., arbitrary units. **(C)** Temperature (T) dependence of the intensity of the ML1 layer line at rest (open circles) and at the plateau of the isometric tetanus (black circles), normalized for the value at rest at 30°C. **(D)** Temperature dependence of the intensity of the AL1 layer line (*I*_AL1_) at the plateau of the isometric tetanus (black circles), normalized for its value at 30°C. The horizontal dashed line represents the expected value of *I*_AL1_ at rest (0.15, see text). Data in C and D are mean ± SEM; six muscles.

The first myosin layer line (ML1, Fig. 2), with an axial spacing of ∼43 nm, close to that of the first myosin meridional reflection (M1; Figs. 2 and S2), is produced by the helical arrangement of the myosin motors on the surface of the thick filaments (Huxley and Brown, 1967) in the OFF state of the thick filament (Linari et al., 2015). The ML1 reflection is strong in the resting state at high temperature and is considerably weakened by either activation or cooling in mammalian muscle (Figs. 2 and 5; Malinchik et al., 1997). Quantitative analysis of the behavior of the ML1 reflection is, however, complicated by the presence of the overlapping first actin layer line (AL1; Fig. 2) from the long-pitched actin helix with axial periodicity ∼37.5 nm in the thin filament (Huxley and Brown, 1967). In resting muscle, when myosin motors are detached from actin, AL1 is too weak to be reliably separated from ML1, but we estimated its contribution as 15% of that observed during active contraction at high temperature as described in the Supplemental text (dashed line in Fig. 5 A). To rigorously exclude any contribution of AL1 to our estimate of the intensity of ML1 (*I*_ML1_), we integrated only the lower-angle part of the reflection as indicated by the vertical dashed lines in Fig. 5 A (Piazzesi et al., 1999). The results show that *I*_ML1_ increases strongly with temperature in resting muscle, at least from 10° to 30°C (Fig. 5 C, open circles). The radial distribution of intensity along the ML1 layer line in resting muscle (Fig. S3) was sampled by the 1,0 and 1,1 equatorial reflections and showed the same strong temperature dependence, with some indication of a shift to lower radial spacing at lower temperature.

In actively contracting muscle, AL1, AL6, and AL7 become stronger (Figs. 2 and 5) as myosin motors attach to actin and a significant fraction of their mass takes up the periodicity of the actin helix (Huxley and Brown, 1967; Koubassova et al., 2008). In these conditions, it was possible to extract the separate intensities of the ML1 and AL1 reflections (*I*_ML1_ and *I*_AL1_) by fitting the axial profile with a double-Gaussian function (Fig. 5 B). The results showed that during active contraction, *I*_ML1_ was a small fraction of its high-temperature resting value, with little dependence on temperature (Fig. 5 C, filled circles). In contrast, *I*_AL1_ increased with increasing temperature, particularly in the lower part of the temperature range (Fig. 5 D), as expected for a higher fraction of myosin motors attached to actin at higher temperature, and consistent with the higher active force (Fig. 1 C) and equatorial intensity ratio (Fig. 3 B).

## Discussion

### The effect of temperature on the x-ray diffraction pattern from resting muscle

Lowering the temperature of mouse EDL muscle from 35° to 10°C produces a general decrease in the intensity of the myosin-based reflections at rest, both meridionals (Fig. S2) and layer lines (Fig. 5, A and C), whereas the intensity of the actin-based layer lines is less dependent on temperature. The effect of lowering the temperature on the myosin-based layer lines has been reported previously for skinned muscle fibers in the relaxed state (Malinchik et al., 1997; Xu et al., 1997) and attributed to a temperature-dependent order-to-disorder transition in the myosin motors. This transition is accompanied by a reduction in the intensity of the 1,0 equatorial reflection and an increase in the equatorial intensity ratio I_1,1_/I_1,0_ (Fig. 3), indicating movement of the motors away from the surface of the thick filament, and an increase in the spacing of the M6 reflection (Fig. 4 H), indicating an increase in thick filament extension. The order-to-disorder transition in the thick filament has been shown to be associated with the nucleotide present in the active site of myosin motor and to the conformation of the motor. At physiological temperature, the equilibrium of ATP hydrolysis is shifted toward its products, the active site pocket is closed, and the motors can take up the helically ordered disposition; at lower temperature, the equilibrium of the hydrolysis step is shifted toward the substrate, the pocket is open, and the motors move away from the surface of the thick filament (Schlichting and Wray, 1986; Málnási-Csizmadia et al., 2000; Xu et al., 2002, 2003, 2009; Takács et al., 2010).

**Figure 6.**
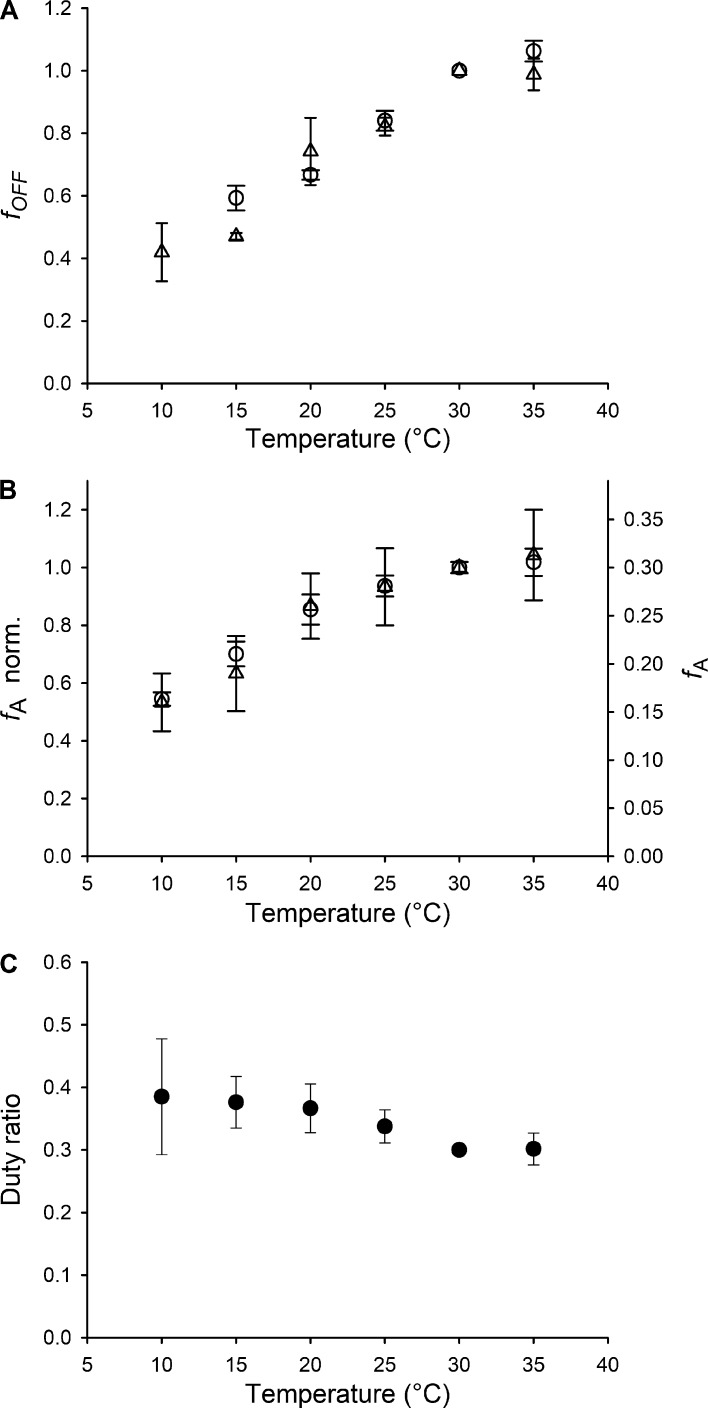
**Fraction of OFF motors at rest and of motors attached in isometric contraction. (A)** Fraction of motors in the OFF (ordered) conformation, *f*_OFF_, at rest as a function of temperature, calculated from the intensity of the M3 (circles, from six muscles) and ML1 (triangles, from three muscles). **(B)** Fraction of motors attached at the plateau of the isometric tetanus, *f*_A_, calculated from the intensity of the M3 (circles, from six muscles) and AL1 (triangles, from three muscles), as described in the text. On the left ordinate axis, *f*_A_ has been normalized to its value at 30°C for a direct comparison with *f*_OFF_ in A. **(C)** Duty ratio (*r*), calculated as explained in the text. Values in A–C are mean ± SEM.

Changes in the intensity of the ML1 layer line and in the intensities and spacings of myosin-based meridional reflections similar to those associated with lowering the temperature of resting muscle occur as a consequence of thick filament mechanosensitivity during active force development, when the number of myosin motors in the helically ordered OFF configuration progressively reduces as a function of the force on the thick filament (Reconditi et al., 2011, 2017; Linari et al., 2015; Piazzesi et al., 2018). In those studies, the number of myosin motors in the OFF state at each time during force development was estimated from the intensities of both the M3 and ML1 reflections, which depend on the fraction of motors in axially (M3) or helically (ML1) ordered conformations. Here too, the fraction of motors in the OFF conformation, *f*_OFF_ (Fig. 6 A, triangles), can be straightforwardly obtained from the square root of the intensity of the ML1 reflection (*I*_ML1_, Fig. 5 C, open circles; Xu et al., 1999; Tsaturyan, 2002). At temperatures <30°C, *f*_OFF_ decreases in proportion to the decrease in temperature, and at 10°C, it becomes 40% of that at near-physiological temperature.

An independent estimate of the change in *f*_OFF_ with temperature can be obtained from *I*_M3_, which is modulated by the number of contributing motors and by their average conformation (Reconditi, 2006). The fine structure of the M3 reflection is largely independent of temperature (apart from the 0.0691-nm^−1^ peak appearing at 10°C; Fig. 4 A, violet), and this indicates that the average conformation of the myosin motors contributing to the M3 is constant over most of the temperature range. The similarity between the x-ray patterns from mouse EDL at rest at 30°C and from frog anterior tibialis at rest at 4°C, in which only the motors in the OFF state contribute to the I_M3_ (Zappe and Maéda, 1985; Reconditi et al., 2011; Linari et al., 2015), suggests that also in the mammalian muscle the disordered detached motors do not contribute to the M3 reflection. Under these conditions, excluding the result at 10°C, the changes in *I*_M3_ at rest in the range of temperatures 15°–35°C only depend on the fraction of motors in the OFF state, *f*_OFF_, which can be estimated from the square root of *I*_M3_ (Fig. 6 A, open circles). The estimates of the temperature dependence of *f*_OFF_ from *I*_M3_ and *I*_ML1_ are in good agreement and indicate that the fraction of motors in the OFF state at 15°C is about half of that at physiological temperature.

### The effect of temperature on the x-ray diffraction pattern during isometric contraction

The force developed at the plateau of an isometric tetanus, *T*_0_, increases monotonically from ∼75 kPa at 10°C to ∼250 kPa at 30°–35°C (Fig. 1). The value of *T*_0_ at physiological temperature is similar to that of frog muscle (a heterothermic animal) at room temperature, but the roughly threefold reduction on cooling to 10°C in mammalian muscle (Ranatunga and Wylie, 1983; Warren et al., 2002) is much larger than that observed in frog muscle on cooling from room temperature to 0°C (∼40%; Linari et al., 2005).

The changes in the x-ray diffraction patterns from rest to isometric contraction show the canonical features reported previously for both frog (Huxley and Brown, 1967; Linari et al., 2000) and mouse (Ma et al., 2018a,b) muscles. The myosin layer lines (Figs. 2, 5, and S3) and the forbidden myosin-based meridional reflections (M1, M2, M4, and M5; Fig. S2) become much weaker, while the M3 and M6 reflections remain strong and their spacings increase (Fig. 4). The fine structure of the M3 reflection changes from a main peak with a small satellite on the HA side to two peaks of similar intensity, while that of the M6 remains almost unchanged. The actin-based layer lines AL1, AL6, and AL7 (Figs. 2 and 5) become stronger as myosin attachment to actin adds mass to the actin helix. The intensity of the 1,0 equatorial reflection decreases while that of the 1,1 increases (Figs. 3 and S1), indicating mass transfer from the thick filaments toward the thin filaments.

**Figure 7.**
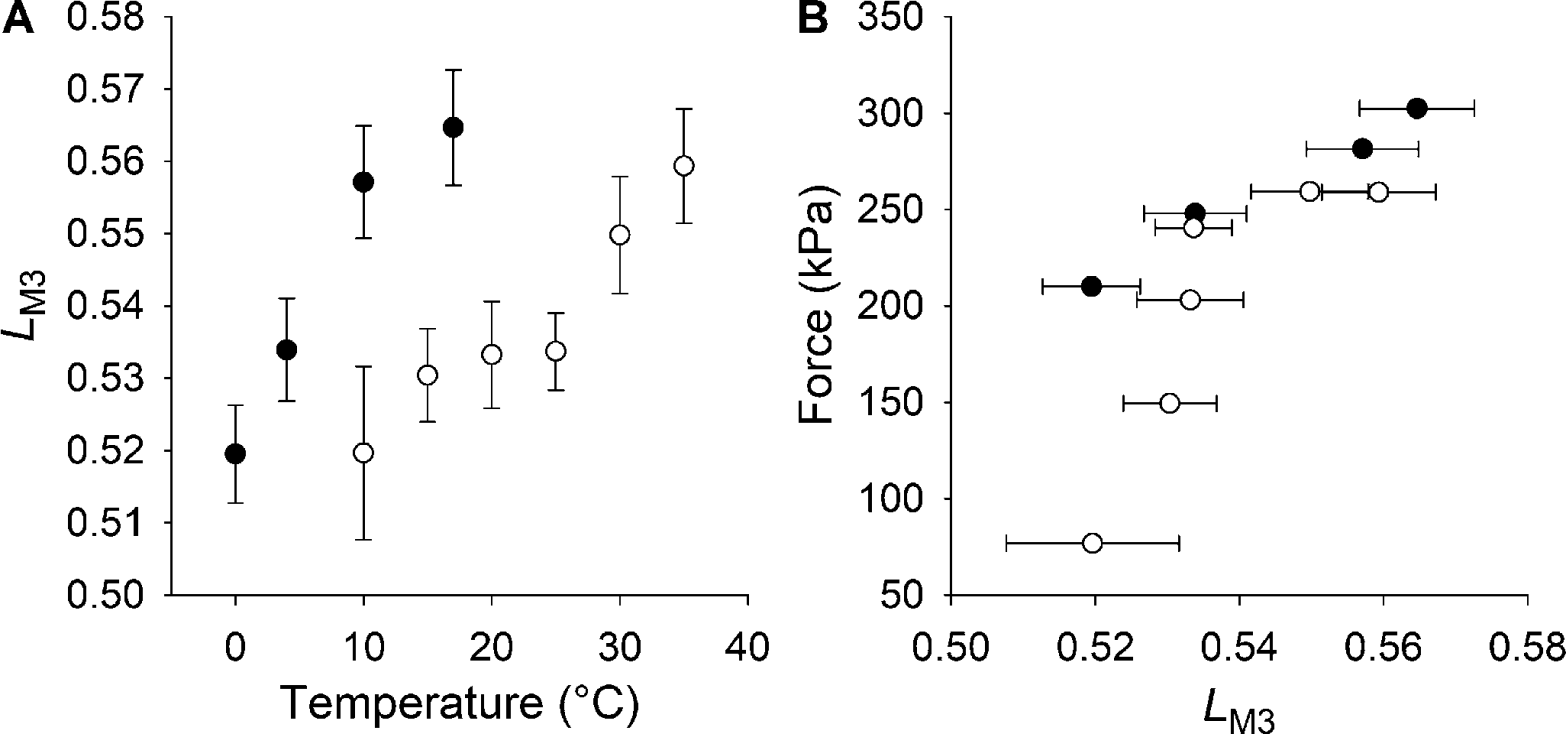
**Comparison of the M3 fine structure in frog and mouse muscles. (A)** Temperature dependence of the M3 fine structure, measured by *L*_M3_ (see text), in frog (filled circles) and mouse (open circles) skeletal muscle. **(B)** Relation between force and *L*_M3_ in isometric tetanic contraction at different temperatures for frog skeletal muscle (filled circles, temperature range 0°–17°C) and for mouse skeletal muscle (open circles, temperature range 10°–35°C). Data are mean ± SEM. The data for frog muscle are from Linari et al. (2005).

The main effects of lowering the temperature on the x-ray patterns recorded during isometric contraction are an approximately threefold decrease of the intensity of the M3 reflection (*I*_M3_, Fig. 4 E) and a twofold reduction in the intensity of the AL1 reflection (Fig. 5 D). The change in *I*_M3_ is accompanied by only a slight change of the M3 fine structure as measured by *L*_M3_, the fractional intensity of the lower-angle peak (LA; Fig. 4 C), which decreases by 8%, from 0.56 to 0.52 (Fig. 4 F). Cooling frog muscle fibers from 17° to 0°C (Linari et al., 2005) induces a similar decrease in *L*_M3_ (Fig. 7 A), but a much smaller reduction in *I*_M3_ (25%), accompanied by a smaller reduction in *T*_0_ (40%), which can be explained by a smaller force per motor, since the number of actin-attached motors is independent of temperature in frog muscle (Ford et al., 1977; Bershitsky et al., 1997; Piazzesi et al., 2003; Decostre et al., 2005). The structural model that reproduces the temperature dependence of the intensity and fine structure of the M3 reflection during active contraction of frog muscle (Linari et al., 2005) cannot reproduce the much larger changes in *I*_M3_ and force observed in the present experiments, which must, therefore, also involve a change in the number of actin-attached motors. For the same change in *L*_M3_ (Fig. 7 A), the changes in force and *I*_M3_ in mouse muscle are more than twice those in frog muscle (Figs. 4 E and 7 B).

The fraction of attached and force-generating motors (*f*_A_) can be calculated from the intensity of the M3 reflection, allowing for the small component associated with the conformational change of the attached motors, as previously described for frog muscle (Irving et al., 2000; Piazzesi et al., 2002; Reconditi et al., 2004; Huxley et al., 2006a). The effects of increasing temperature from 10° to 35°C can be explained by an axial motion of the catalytic domain of the motors by 1.4 nm toward the center of the sarcomere, associated with progression of the working stroke in the motor (Linari et al., 2005). This makes only a small contribution to the increase in *I*_M3_, ∼15%, compared with the observed threefold change. The rest of the increase in *I*_M3_ can be explained by an increase in the number of attached motors. Fig. 6 B shows *f*_A_ calculated from *I*_M3_ (circles) plotted both as a fraction of the motors attached at physiological temperature (left ordinate) and as a fraction of the total number of motors per half-thick filament (right ordinate), assumed to be 30% at near physiological temperature. This assumption is based on measurements on Ca^2+^-activated skinned fibers from rabbit psoas muscle (Linari et al., 2007). In those experiments, the fraction of motors responsible for the generation of isometric force was determined by comparing half-sarcomere stiffness in active isometric contraction and in rigor (the ATP-depleted state in which all motors are attached to actin; Cooke and Franks, 1980; Lovell et al., 1981). An independent estimate of the effect of temperature on *f*_A_ can be obtained from the change in intensity of the AL1 layer line (Fig. 5 D), using the structural model of the actin-myosin complex of Koubassova et al. (2008) (see Supplemental text and Figs. S4 and S5), which takes into account the contributions of the actin monomers and of the myosin motors stereospecifically attached to actin (Fig. 6 B, triangles) and, unlike AL6 and AL7, is not sensitive to the tilting of the light chain domain of the attached motors. The *f*_A_-temperature relations independently estimated from *I*_M3_ and *I*_AL1_ are in good agreement: in both cases, *f*_A_ is halved at 10°–15°C.

### The nature of the disordered state populated by lowering temperature at rest

The most striking result of the above analysis is that decreasing the temperature from the near-physiological value (30°–35°C) to 10°C produces a parallel twofold decrease in the fraction of both the motors in the OFF state at rest (*f*_OFF_) and the motors in the attached, force-generating state at *T*_0_ (*f*_A_). This suggests that the fraction of motors that are in the OFF state at rest is equal to the fraction that becomes available for actin attachment (switched ON) on activation. If, at any temperature, *f*_OFF_ also measures the fraction *f*_ON_ of motors that can be recruited (switched ON) upon activation for interaction with actin, while the disordered fraction is unavailable for attachment, then *f*_A_/*f*_OFF_ (= *f*_A_/*f*_ON_) measures the duty ratio *r*, i.e., the fraction of the ATPase cycle spent by a motor in the attached force-generating state. *r*, 0.3 at physiological temperature at which all the myosin motors are switched ON during a tetanic contraction (Linari et al., 2015), remains almost constant over the full temperature range studied here (Fig. 6 C). It follows that, in mammalian skeletal muscle, reduction of temperature below the normal physiological value has a regulatory function, reducing the number of motors available for actin interaction, without changing *r*, in agreement with the previous finding for muscle fibers of the heterothermic frog (Piazzesi et al., 2003), in which all motors are available independent of temperature. A constant value of *r* in isometric contraction is expected for a mechano-enzyme like myosin, for which the rate constant for detachment from actin depends on strain (Huxley, 1957), so *r* would change only in response to a change of load or shortening velocity.

The existence of a disordered refractory population of motors at low temperature is also qualitatively supported by the observation that the reduction in the intensity of the 1,0 equatorial reflection going from rest to *T*_0_ also decreases with temperature (Fig. 3 A). The high value of *I*_1,0_ in resting muscle at high temperature is associated with motors lying on the surface of the thick filament in their OFF state; at lower temperatures, the motors move away from the filament surface, so fewer motors are left to be switched ON by activation.

**Figure 8.**
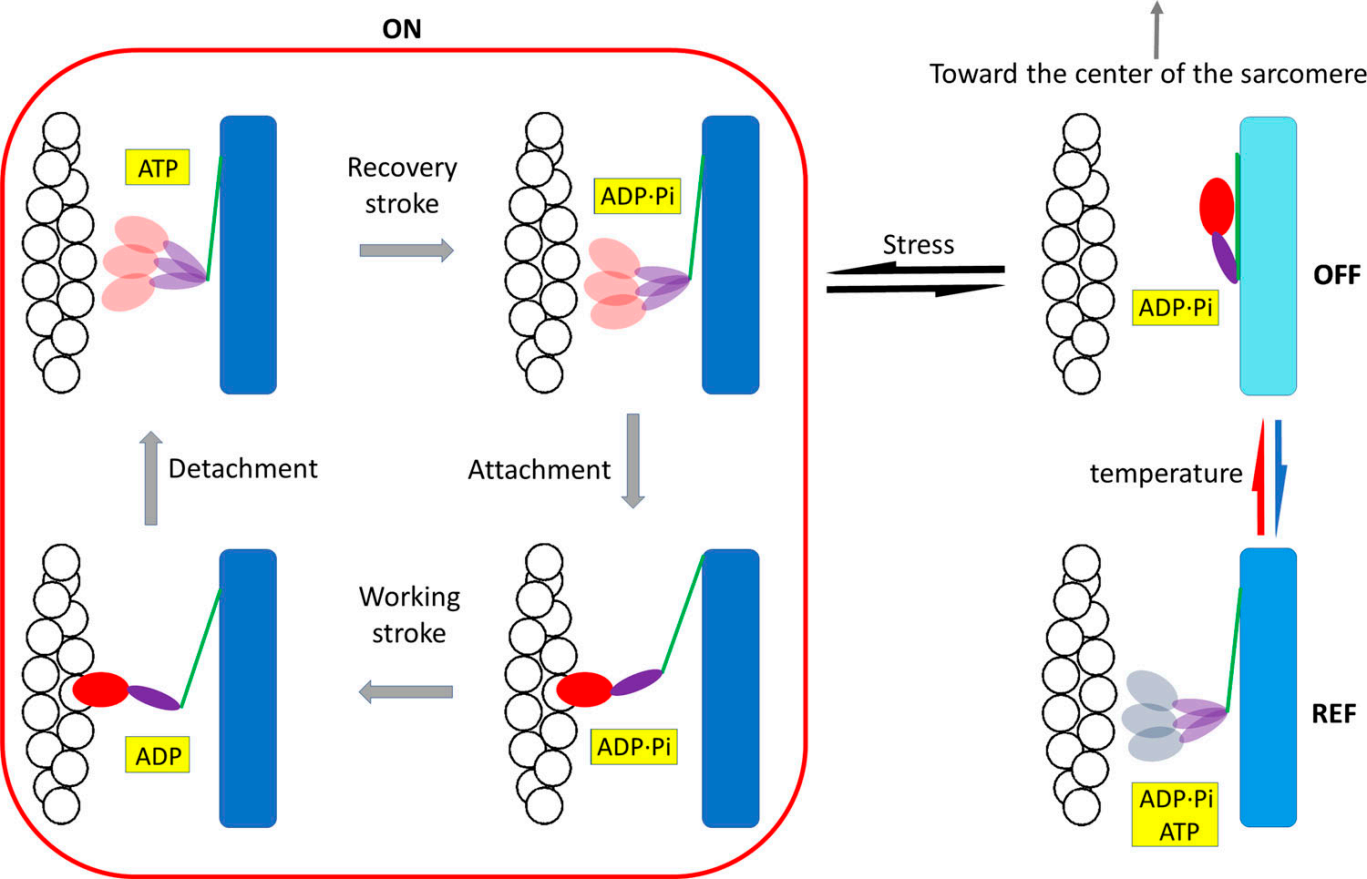
**Scheme of the regulatory effect of temperature on thick filament.** Myosin motors at rest either lie on the surface of the thick filament in the ordered OFF state, folded back toward the center of the sarcomere, or are in a disordered state refractory to activation, REF. Lowering the temperature (blue arrow) favors the REF state, while increasing the temperature (red arrow) favors the OFF state. Upon muscle activation, only the fraction of motors in the OFF state are recruited, in relation to the stress on the thick filament (black arrows), into the disordered ON ADP.Pi-state, from which they can attach to actin and enter the mechanochemical cycle (bounded by the red line). The state of the ligand in the nucleotide-binding pocket of the motor is highlighted in yellow. The motor domain of the head is red except in the REF state (gray), for which the state of the ligand is not defined. The backbone of the thick filament has a shorter periodicity in the OFF state (cyan), which increases both in the REF state (light blue) or when the thick filament is switched ON (blue) because motors have moved away from the ordered helical disposition along the surface of the filament.

The regulatory action of temperature in mammalian skeletal muscle revealed in this study is integrated into the conventional mechanochemical cycle with additional steps of thick filament regulation in Fig. 8. At near-physiological temperature, myosin motors at rest lie on the surface of the thick filament in the OFF (ordered) state and, during a loaded contraction, are recruited by the stress on the thick filament into the disordered ON state, from which they can attach to actin and enter into the mechanochemical cycle that implies the execution of the working stroke, followed by ATP-driven detachment, the hydrolysis step, and reversal of the working stroke. The load on the muscle during contraction and thus the stress on the thick filament determine whether after detachment the motors with ADP-Pi in the pocket remain in the ON state or return to the OFF state (Linari et al., 2015; Fusi et al., 2017). Lowering the temperature of the resting muscle (blue arrow in Fig. 8) reduces the population of the motors in the OFF state, presumably by weakening the intramolecular/intermolecular interactions responsible for the maintenance of the OFF state, so motors move away from the ordered disposition along the surface of the thick filament, attaining a disordered conformation that differs from the ON state in that it is refractory to activation (REF).

The underlying structural changes could be accompanied by opening of the nucleotide binding pocket and a shift in the equilibrium of the hydrolysis step toward ATP. However, the finding that temperature affects the conformation of the myosin head independent of the state of the nucleotide in the pocket (Xu et al., 2003) suggests that the present x-ray data cannot define the biochemical state of the head in the REF state; the pocket may contain ATP or ADP.Pi (Fig. 8). In skinned mammalian muscle fibers, lowering temperature favors a disordered ATP state (Xu et al., 2002), but membrane permeabilization induces a partial loss of the regulatory role of temperature. A decrease in temperature from ∼20° to 10°C, which reduced *T*_0_ by 60% in the present experiments, reduced *T*_0_ by only 30% in skinned fibers from rabbit psoas muscle, without significant effects on the stiffness and thus on the number of attached motors (Linari et al., 2007). Unless there is a species-specific difference in the temperature sensitivity of the thick filament state, this difference indicates that the effect of temperature on the equilibrium of the hydrolysis defined in relaxed skinned fibers is not a reliable indicator of the state of the nucleotide in the intact muscle at rest. Accordingly, the order-to-disorder transition induced by lowering temperature in relaxed skinned fibers does not affect the number of motors available for actin attachment upon Ca^2+^ activation.

The identification in intact mammalian muscle of a new disordered state refractory to activation indicates that structural and functional definitions of the OFF and ON states no longer coincide. Two structurally different types of the myosin OFF state exist, both unable to attach to actin and split ATP: an OFF-ordered state, which can be switched ON upon activation, and an OFF-disordered state or REF state, populated at low temperature, which is refractory to activation. In the OFF-ordered state, myosin motors lie along helical tracks on the surface of the thick filament, and this structure is stabilized by both intramolecular interactions (head-head and head-tail of the same dimer) and intermolecular interactions (head and tail of one molecule interacting with other myosin molecules and with MyBP-C and titin; Trivedi et al., 2018). Lowering the temperature in mammalian muscle may promote the transition to the REF state by disrupting the interactions responsible for both the ordered state and its switchability, while preserving the interactions that inhibit actin attachment and ATP hydrolysis. Considering that the inhibited state of the myosin molecule is primarily characterized as the IHM state, the conserved structure responsible for myosin inhibition also seen in nonmuscle myosin II, independent of the regulatory mechanism (Lee et al., 2018), some features of the IHM are likely to be conserved in the REF state attained at low temperature. Under this condition, the intermolecular interactions generated in striated muscle by the polymeric organization of myosin in the thick filament and with MyBP-C and titin are likely to constitute the regulatory elements that control the disruption of the IHM state and switching ON of the motors. Lowering the temperature could specifically affect the intermolecular interactions, blunting both their control of the IHM state, that becomes refractory, and the ordered disposition of myosin heads along the thick filament. In this scenario, the REF-disordered state may structurally differ from the ON state by the preservation of the IHM features that inhibit actin attachment and ATP hydrolysis. To test this hypothesis, the biochemical and structural differences between the REF and ON states must be investigated in detail.

The cooling-dependent accumulation of myosin motors of mammalian muscle in a refractory state in which they are unavailable for actin interaction could be an energetically convenient regulatory mechanism for mammals that undergo hibernation. This is the physiological state in which body temperature drops to near ambient and metabolic demand decreases as vegetative activities, such as heart and respiration rates, slow drastically. Hibernation implies mechanisms that contribute to metabolic depression by global suppression of ATP-expensive reactions as posttranslational modification of proteins and enzymes (Storey, 2010). Since muscles contribute ∼40% to body mass, a regulatory mechanism that at low temperature reduces ATP utilization by skeletal muscles, irrespective of residual motoneuron activity, would represent an efficient modulator of whole-body energy consumption during hibernation of mammals.

## Supplementary Material

Supplemental Materials (PDF)

## References

[bib1] Bershitsky S.Y., Tsaturyan A.K., Bershitskaya O.N., Mashanov G.I., Brown P., Burns R., and Ferenczi M.A. 1997 Muscle force is generated by myosin heads stereospecifically attached to actin. Nature. 388:186–190. 10.1038/40651 9217160

[bib2] Bordas J., Lowy J., Svensson A., Harries J.E., Diakun G.P., Gandy J., Miles C., Mant G.R., and Towns-Andrews E. 1995 X-ray evidence that in contracting live frog muscles there exist two distinct populations of myosin heads. Biophys. J. 68(4 Suppl):99S–104S. 7787116 PMC1281885

[bib3] Brenner B., and Yu L.C. 1991 Characterization of radial force and radial stiffness in Ca(2+)-activated skinned fibres of the rabbit psoas muscle. J. Physiol. 441:703–718. 10.1113/jphysiol.1991.sp018774 1816390 PMC1180221

[bib4] Brunello E., Fusi L., Reconditi M., Linari M., Bianco P., Panine P., Narayanan T., Piazzesi G., Lombardi V., and Irving M. 2009 Structural changes in myosin motors and filaments during relaxation of skeletal muscle. J. Physiol. 587:4509–4521. 10.1113/jphysiol.2009.176222 19651765 PMC2766654

[bib5] Burkholder T.J., Fingado B., Baron S., and Lieber R.L. 1994 Relationship between muscle fiber types and sizes and muscle architectural properties in the mouse hindlimb. J. Morphol. 221:177–190. 10.1002/jmor.1052210207 7932768

[bib6] Cecchi G., Bagni M.A., Griffiths P.J., Ashley C.C., and Maeda Y. 1990 Detection of radial crossbridge force by lattice spacing changes in intact single muscle fibers. Science. 250:1409–1411. 10.1126/science.2255911 2255911

[bib7] Close R.I. 1972 Dynamic properties of mammalian skeletal muscles. Physiol. Rev. 52:129–197. 10.1152/physrev.1972.52.1.129 4256989

[bib8] Colson B.A., Locher M.R., Bekyarova T., Patel J.R., Fitzsimons D.P., Irving T.C., and Moss R.L. 2010 Differential roles of regulatory light chain and myosin binding protein-C phosphorylations in the modulation of cardiac force development. J. Physiol. 588:981–993. 10.1113/jphysiol.2009.183897 20123786 PMC2849963

[bib9] Colson B.A., Patel J.R., Chen P.P., Bekyarova T., Abdalla M.I., Tong C.W., Fitzsimons D.P., Irving T.C., and Moss R.L. 2012 Myosin binding protein-C phosphorylation is the principal mediator of protein kinase A effects on thick filament structure in myocardium. J. Mol. Cell. Cardiol. 53:609–616. 10.1016/j.yjmcc.2012.07.012 22850286 PMC3472100

[bib10] Cooke R., and Franks K. 1980 All myosin heads form bonds with actin in rigor rabbit skeletal muscle. Biochemistry. 19:2265–2269. 10.1021/bi00551a042 6103713

[bib11] Decostre V., Bianco P., Lombardi V., and Piazzesi G. 2005 Effect of temperature on the working stroke of muscle myosin. Proc. Natl. Acad. Sci. USA. 102:13927–13932. 10.1073/pnas.0506795102 16172377 PMC1236584

[bib12] Ebashi S., Endo M., and Otsuki I. 1969 Control of muscle contraction. Q. Rev. Biophys. 2:351–384. 10.1017/S0033583500001190 4935801

[bib13] Fischetti R., Stepanov S., Rosenbaum G., Barrea R., Black E., Gore D., Heurich R., Kondrashkina E., Kropf A.J., Wang S., 2004 The BioCAT undulator beamline 18ID: a facility for biological non-crystalline diffraction and X-ray absorption spectroscopy at the Advanced Photon Source. J. Synchrotron Radiat. 11:399–405. 10.1107/S0909049504016760 15310956

[bib14] Ford L.E., Huxley A.F., and Simmons R.M. 1977 Tension responses to sudden length change in stimulated frog muscle fibres near slack length. J. Physiol. 269:441–515. 10.1113/jphysiol.1977.sp011911 302333 PMC1283722

[bib15] Fusi L., Percario V., Brunello E., Caremani M., Bianco P., Powers J.D., Reconditi M., Lombardi V., and Piazzesi G. 2017 Minimum number of myosin motors accounting for shortening velocity under zero load in skeletal muscle. J. Physiol. 595:1127–1142. 10.1113/JP273299 27763660 PMC5309372

[bib16] Gordon A.M., Homsher E., and Regnier M. 2000 Regulation of contraction in striated muscle. Physiol. Rev. 80:853–924. 10.1152/physrev.2000.80.2.853 10747208

[bib17] Hammersley A. 2016 FIT2D: a multi-purpose data reduction, analysis and visualization program. J. Appl. Cryst. 49:646–652. 10.1107/S1600576716000455

[bib18] Haselgrove J.C. 1975 X-ray evidence for conformational changes in the myosin filaments of vertebrate striated muscle. J. Mol. Biol. 92:113–143. 10.1016/0022-2836(75)90094-7 1080204

[bib19] Haselgrove J.C., and Huxley H.E. 1973 X-ray evidence for radial cross-bridge movement and for the sliding filament model in actively contracting skeletal muscle. J. Mol. Biol. 77:549–568. 10.1016/0022-2836(73)90222-2 4541885

[bib20] Herron T.J., Korte F.S., and McDonald K.S. 2001 Power output is increased after phosphorylation of myofibrillar proteins in rat skinned cardiac myocytes. Circ. Res. 89:1184–1190. 10.1161/hh2401.101908 11739284

[bib21] Hooijman P., Stewart M.A., and Cooke R. 2011 A new state of cardiac myosin with very slow ATP turnover: a potential cardioprotective mechanism in the heart. Biophys. J. 100:1969–1976. 10.1016/j.bpj.2011.02.061 21504733 PMC3077696

[bib22] Houdusse A., Kalabokis V.N., Himmel D., Szent-Györgyi A.G., and Cohen C. 1999 Atomic structure of scallop myosin subfragment S1 complexed with MgADP: a novel conformation of the myosin head. Cell. 97:459–470. 10.1016/S0092-8674(00)80756-4 10338210

[bib23] Huxley A.F. 1957 Muscle structure and theories of contraction. Prog. Biophys. Biophys. Chem. 7:255–318. 10.1016/S0096-4174(18)30128-8 13485191

[bib24] Huxley A.F. 1973 A note suggesting that the cross-bridge attachment during muscle contraction may take place in two stages. Proc. R. Soc. Lond. B Biol. Sci. 183:83–86. 10.1098/rspb.1973.0006 4144558

[bib25] Huxley H.E., and Brown W. 1967 The low-angle x-ray diagram of vertebrate striated muscle and its behaviour during contraction and rigor. J. Mol. Biol. 30:383–434. 10.1016/S0022-2836(67)80046-9 5586931

[bib26] Huxley H.E., Faruqi A.R., Kress M., Bordas J., and Koch M.H. 1982 Time-resolved X-ray diffraction studies of the myosin layer-line reflections during muscle contraction. J. Mol. Biol. 158:637–684. 10.1016/0022-2836(82)90253-4 6981706

[bib27] Huxley H.E., Simmons R.M., Faruqi A.R., Kress M., Bordas J., and Koch M.H. 1983 Changes in the X-ray reflections from contracting muscle during rapid mechanical transients and their structural implications. J. Mol. Biol. 169:469–506. 10.1016/S0022-2836(83)80062-X 6604821

[bib28] Huxley H., Reconditi M., Stewart A., and Irving T. 2006 a X-ray interference studies of crossbridge action in muscle contraction: evidence from muscles during steady shortening. J. Mol. Biol. 363:762–772. 10.1016/j.jmb.2006.08.055 16979661

[bib29] Huxley H., Reconditi M., Stewart A., and Irving T. 2006 b X-ray interference studies of crossbridge action in muscle contraction: evidence from quick releases. J. Mol. Biol. 363:743–761. 10.1016/j.jmb.2006.08.075 17007871

[bib30] Irving M. 2017 Regulation of Contraction by the Thick Filaments in Skeletal Muscle. Biophys. J. 113:2579–2594. 10.1016/j.bpj.2017.09.037 29262355 PMC5770512

[bib31] Irving M., Piazzesi G., Lucii L., Sun Y.B., Harford J.J., Dobbie I.M., Ferenczi M.A., Reconditi M., and Lombardi V. 2000 Conformation of the myosin motor during force generation in skeletal muscle. Nat. Struct. Biol. 7:482–485. 10.1038/75890 10881196 PMC8397617

[bib32] Kampourakis T., Sun Y.B., and Irving M. 2016 Myosin light chain phosphorylation enhances contraction of heart muscle via structural changes in both thick and thin filaments. Proc. Natl. Acad. Sci. USA. 113:E3039–E3047. 10.1073/pnas.1602776113 27162358 PMC4889392

[bib33] Koubassova N.A., Bershitsky S.Y., Ferenczi M.A., and Tsaturyan A.K. 2008 Direct modeling of X-ray diffraction pattern from contracting skeletal muscle. Biophys. J. 95:2880–2894. 10.1529/biophysj.107.120832 18539638 PMC2527261

[bib34] Lee K.H., Sulbarán G., Yang S., Mun J.Y., Alamo L., Pinto A., Sato O., Ikebe M., Liu X., Korn E.D., 2018 Interacting-heads motif has been conserved as a mechanism of myosin II inhibition since before the origin of animals. Proc. Natl. Acad. Sci. USA. 115:E1991–E2000. 10.1073/pnas.1715247115 29444861 PMC5834683

[bib35] Linari M., Piazzesi G., Dobbie I., Koubassova N., Reconditi M., Narayanan T., Diat O., Irving M., and Lombardi V. 2000 Interference fine structure and sarcomere length dependence of the axial x-ray pattern from active single muscle fibers. Proc. Natl. Acad. Sci. USA. 97:7226–7231. 10.1073/pnas.97.13.7226 10860988 PMC16527

[bib36] Linari M., Brunello E., Reconditi M., Sun Y.B., Panine P., Narayanan T., Piazzesi G., Lombardi V., and Irving M. 2005 The structural basis of the increase in isometric force production with temperature in frog skeletal muscle. J. Physiol. 567:459–469. 10.1113/jphysiol.2005.089672 15961426 PMC1474186

[bib37] Linari M., Caremani M., Piperio C., Brandt P., and Lombardi V. 2007 Stiffness and fraction of Myosin motors responsible for active force in permeabilized muscle fibers from rabbit psoas. Biophys. J. 92:2476–2490. 10.1529/biophysj.106.099549 17237201 PMC1864836

[bib38] Linari M., Brunello E., Reconditi M., Fusi L., Caremani M., Narayanan T., Piazzesi G., Lombardi V., and Irving M. 2015 Force generation by skeletal muscle is controlled by mechanosensing in myosin filaments. Nature. 528:276–279. 10.1038/nature15727 26560032

[bib39] Lovell S.J., Knight P.J., and Harrington W.F. 1981 Fraction of myosin heads bound to thin filaments in rigor fibrils from insect flight and vertebrate muscles. Nature. 293:664–666. 10.1038/293664a0 7290203

[bib40] Lowy J., Popp D., and Stewart A.A. 1991 X-ray studies of order-disorder transitions in the myosin heads of skinned rabbit psoas muscles. Biophys. J. 60:812–824. 10.1016/S0006-3495(91)82116-6 1742454 PMC1260133

[bib41] Ma W., Gong H., and Irving T. 2018 a Myosin Head Configurations in Resting and Contracting Murine Skeletal Muscle. Int. J. Mol. Sci. 19:2643 10.3390/ijms19092643 PMC6165214 30200618

[bib42] Ma W., Gong H., Kiss B., Lee E.J., Granzier H., and Irving T. 2018 b Thick-Filament Extensibility in Intact Skeletal Muscle. Biophys. J. 115:1580–1588. 10.1016/j.bpj.2018.08.038 30266320 PMC6196444

[bib43] Malinchik S.B., and Lednev V.V. 1992 Interpretation of the X-ray diffraction pattern from relaxed skeletal muscle and modelling of the thick filament structure. J. Muscle Res. Cell Motil. 13:406–419. 10.1007/BF01738036 1401037

[bib44] Malinchik S., Xu S., and Yu L.C. 1997 Temperature-induced structural changes in the myosin thick filament of skinned rabbit psoas muscle. Biophys. J. 73:2304–2312. 10.1016/S0006-3495(97)78262-6 9370427 PMC1181135

[bib45] Málnási-Csizmadia A., Woolley R.J., and Bagshaw C.R. 2000 Resolution of conformational states of Dictyostelium myosin II motor domain using tryptophan (W501) mutants: implications for the open-closed transition identified by crystallography. Biochemistry. 39:16135–16146. 10.1021/bi001125j 11123942

[bib46] Málnási-Csizmadia A., Pearson D.S., Kovács M., Woolley R.J., Geeves M.A., and Bagshaw C.R. 2001 Kinetic resolution of a conformational transition and the ATP hydrolysis step using relaxation methods with a Dictyostelium myosin II mutant containing a single tryptophan residue. Biochemistry. 40:12727–12737. 10.1021/bi010963q 11601998

[bib47] Moos C., Mason C.M., Besterman J.M., Feng I.N., and Dubin J.H. 1978 The binding of skeletal muscle C-protein to F-actin, and its relation to the interaction of actin with myosin subfragment-1. J. Mol. Biol. 124:571–586. 10.1016/0022-2836(78)90172-9 152359

[bib48] Narayanan T., Sztucki M., Van Vaerenbergh P., Léonardon J., Gorini J., Claustre L., Sever F., Morse J., and Boesecke P. 2018 A multipurpose instrument for time-resolved ultra-small-angle and coherent X-ray scattering. J. Appl. Cryst. 51:1511–1524. 10.1107/S1600576718012748 30546286 PMC6276275

[bib49] Piazzesi G., Reconditi M., Dobbie I., Linari M., Boesecke P., Diat O., Irving M., and Lombardi V. 1999 Changes in conformation of myosin heads during the development of isometric contraction and rapid shortening in single frog muscle fibres. J. Physiol. 514:305–312. 10.1111/j.1469-7793.1999.305ae.x 9852315 PMC2269081

[bib50] Piazzesi G., Reconditi M., Linari M., Lucii L., Sun Y.B., Narayanan T., Boesecke P., Lombardi V., and Irving M. 2002 Mechanism of force generation by myosin heads in skeletal muscle. Nature. 415:659–662. 10.1038/415659a 11832949

[bib51] Piazzesi G., Reconditi M., Koubassova N., Decostre V., Linari M., Lucii L., and Lombardi V. 2003 Temperature dependence of the force-generating process in single fibres from frog skeletal muscle. J. Physiol. 549:93–106. 10.1113/jphysiol.2002.038703 12665607 PMC2342933

[bib52] Piazzesi G., Caremani M., Linari M., Reconditi M., and Lombardi V. 2018 Thick Filament Mechano-Sensing in Skeletal and Cardiac Muscles: A Common Mechanism Able to Adapt the Energetic Cost of the Contraction to the Task. Front. Physiol. 9:736 10.3389/fphys.2018.00736 29962967 PMC6010558

[bib53] Ranatunga K.W., and Wylie S.R. 1983 Temperature-dependent transitions in isometric contractions of rat muscle. J. Physiol. 339:87–95. 10.1113/jphysiol.1983.sp014704 6887040 PMC1199149

[bib54] Reconditi M. 2006 Recent improvements in small angle x-ray diffraction for the study of muscle physiology. Rep. Prog. Phys. 69:2709–2759. 10.1088/0034-4885/69/10/R01 19946470 PMC2783642

[bib55] Reconditi M., Linari M., Lucii L., Stewart A., Sun Y.B., Boesecke P., Narayanan T., Fischetti R.F., Irving T., Piazzesi G., 2004 The myosin motor in muscle generates a smaller and slower working stroke at higher load. Nature. 428:578–581. 10.1038/nature02380 15058307

[bib56] Reconditi M., Brunello E., Linari M., Bianco P., Narayanan T., Panine P., Piazzesi G., Lombardi V., and Irving M. 2011 Motion of myosin head domains during activation and force development in skeletal muscle. Proc. Natl. Acad. Sci. USA. 108:7236–7240. 10.1073/pnas.1018330108 21482782 PMC3084075

[bib57] Reconditi M., Brunello E., Fusi L., Linari M., Martinez M.F., Lombardi V., Irving M., and Piazzesi G. 2014 Sarcomere-length dependence of myosin filament structure in skeletal muscle fibres of the frog. J. Physiol. 592:1119–1137. 10.1113/jphysiol.2013.267849 24344169 PMC3948567

[bib58] Reconditi M., Caremani M., Pinzauti F., Powers J.D., Narayanan T., Stienen G.J., Linari M., Lombardi V., and Piazzesi G. 2017 Myosin filament activation in the heart is tuned to the mechanical task. Proc. Natl. Acad. Sci. USA. 114:3240–3245. 10.1073/pnas.1619484114 28265101 PMC5373356

[bib59] Schlichting I., and Wray J. 1986 Behaviour of crossbridges in non-overlap frog muscle in the presence and absence of ATP. J. Muscle Res. Cell Motil. 7:79–80.

[bib60] Stewart M.A., Franks-Skiba K., Chen S., and Cooke R. 2010 Myosin ATP turnover rate is a mechanism involved in thermogenesis in resting skeletal muscle fibers. Proc. Natl. Acad. Sci. USA. 107:430–435. 10.1073/pnas.0909468107 19966283 PMC2806748

[bib61] Storey K.B. 2010 Out cold: biochemical regulation of mammalian hibernation - a mini-review. Gerontology. 56:220–230. 10.1159/000228829 19602865

[bib62] Takács B., Billington N., Gyimesi M., Kintses B., Málnási-Csizmadia A., Knight P.J., and Kovács M. 2010 Myosin complexed with ADP and blebbistatin reversibly adopts a conformation resembling the start point of the working stroke. Proc. Natl. Acad. Sci. USA. 107:6799–6804. 10.1073/pnas.0907585107 20351242 PMC2872372

[bib63] Trivedi D.V., Adhikari A.S., Sarkar S.S., Ruppel K.M., and Spudich J.A. 2018 Hypertrophic cardiomyopathy and the myosin mesa: viewing an old disease in a new light. Biophys. Rev. 10:27–48. 10.1007/s12551-017-0274-6 28717924 PMC5803174

[bib64] Tsaturyan A.K. 2002 Diffraction by partially occupied helices. Acta Crystallogr. A. 58:292–294. 10.1107/S0108767302001307 11961291

[bib65] Warren G.L., Ingalls C.P., and Armstrong R.B. 2002 Temperature dependency of force loss and Ca(2+) homeostasis in mouse EDL muscle after eccentric contractions. Am. J. Physiol. Regul. Integr. Comp. Physiol. 282:R1122–R1132. 10.1152/ajpregu.00671.2001 11893617

[bib66] Williams C.D., Regnier M., and Daniel T.L. 2010 Axial and radial forces of cross-bridges depend on lattice spacing. PLOS Comput. Biol. 6:e1001018 10.1371/journal.pcbi.1001018 21152002 PMC2996315

[bib67] Woodhead J.L., Zhao F.Q., Craig R., Egelman E.H., Alamo L., and Padrón R. 2005 Atomic model of a myosin filament in the relaxed state. Nature. 436:1195–1199. 10.1038/nature03920 16121187

[bib68] Wray J.S. 1987 Structure of relaxed myosin filaments in relation to nucleotide state in vertebrate skeletal muscle. J. Muscle Res. Cell Motil. 8:62.

[bib69] Xu S., Malinchik S., Gilroy D., Kraft T., Brenner B., and Yu L.C. 1997 X-ray diffraction studies of cross-bridges weakly bound to actin in relaxed skinned fibers of rabbit psoas muscle. Biophys. J. 73:2292–2303. 10.1016/S0006-3495(97)78261-4 9370426 PMC1181134

[bib70] Xu S., Gu J., Rhodes T., Belknap B., Rosenbaum G., Offer G., White H., and Yu L.C. 1999 The M.ADP.Pi state is required for helical order in the thick filaments of skeletal muscle. Biophys. J. 77:2665–2676. 10.1016/S0006-3495(99)77101-8 10545367 PMC1300541

[bib71] Xu S., Gu J., Melvin G., and Yu L.C. 2002 Structural characterization of weakly attached cross-bridges in the A*M*ATP state in permeabilized rabbit psoas muscle. Biophys. J. 82:2111–2122. 10.1016/S0006-3495(02)75558-6 11916867 PMC1302005

[bib72] Xu S., Offer G., Gu J., White H.D., and Yu L.C. 2003 Temperature and ligand dependence of conformation and helical order in myosin filaments. Biochemistry. 42:390–401. 10.1021/bi026085t 12525166

[bib73] Xu S., Martyn D., Zaman J., and Yu L.C. 2006 X-ray diffraction studies of the thick filament in permeabilized myocardium from rabbit. Biophys. J. 91:3768–3775. 10.1529/biophysj.106.088971 16950853 PMC1630466

[bib74] Xu S., White H.D., Offer G.W., and Yu L.C. 2009 Stabilization of helical order in the thick filaments by blebbistatin: further evidence of coexisting multiple conformations of myosin. Biophys. J. 96:3673–3681. 10.1016/j.bpj.2009.01.049 19413972 PMC2711421

[bib75] Zappe H.A., and Maéda Y. 1985 X-ray diffraction study of fast and slow mammalian skeletal muscle in the live relaxed state. J. Mol. Biol. 185:211–214. 10.1016/0022-2836(85)90193-7 4046039

[bib76] Zoghbi M.E., Woodhead J.L., Moss R.L., and Craig R. 2008 Three-dimensional structure of vertebrate cardiac muscle myosin filaments. Proc. Natl. Acad. Sci. USA. 105:2386–2390. 10.1073/pnas.0708912105 18252826 PMC2268146

