# Supplementary material for: Low temperature traps myosin motors of mammalian muscle in a refractory state that prevents activation
Source: J Gen Physiol. 2019 Sep 25;151(11):1272–86. doi: 10.1085/jgp.201912424 (PMC6829559; doi:10.1085/jgp.201912424)
Supplement: Supplemental Materials (PDF) [file JGP_201912424_sm.pdf]

Supplemental material

Caremani et al., https://doi.org/10.1085/jgp.201912424

The fraction of motors attached to actin in isometric contraction

The fraction of actin-attached motors at T_0 at 30°C is assumed to be 0.3 based on measurements on Ca^{2+}-activated skinned fibers from rabbit psoas at 12°C (Linari et al., 2007). In the same experiments, it was shown that changes in temperature in the range of 5°–20°C do not change the fraction of attached motors, but only the force generated per motor. This is in apparent contradiction with the finding in this paper that in intact mammalian muscle, cooling reduces the force in isometric contraction by reducing both the force per motor and the number of attached motors. The difference could be explained by considering that part of the regulatory effect of temperature on the thick filament may be lost on permeabilization of the surface membrane (see Discussion).

Estimate of the fraction of attached motors from the intensity of the first actin-based layer line

To determine the temperature dependence of the fraction of attached motors (f_A) at the plateau of the isometric tetanus from I_{AL1}, we compared the present data with the f_A-versus-I_{AL1} relation in the model of Koubassova et al. (2008) (Fig. 6 A in that paper). The model f_A–I_{AL1} relation (Fig. S4) was normalized so that I_{AL1} = 1 for f_A = 1 (corresponding to the rigor condition, with all the motors attached to actin).

To calculate f_A, we first assumed a value ($f_{A,30}$) for the fraction of attached motors at 30°C, then scaled I_{AL1} at the various temperatures to that predicted by the model for the assumed $f_{A,30}$. Then we used the model to calculate f_A at the other temperatures from the scaled I_{AL1} values.

Fig. S5 A shows the temperature dependence of f_A for different assumptions: $f_{A,30}$ = 0.2 black, 0.3 red, 0.4 green. In Fig. S5 B, the same relations (triangles with the same color code as in A) are normalized to $f_{A,30}$, showing that the fractional change predicted by the model is quite independent of the assumption about the fraction of attached motors at 30°C. The temperature dependence of f_A calculated using the intensity of the M3 reflection as described in the main text (circles from Fig. 6 B in Koubassova et al., 2008) is superimposed for comparison.

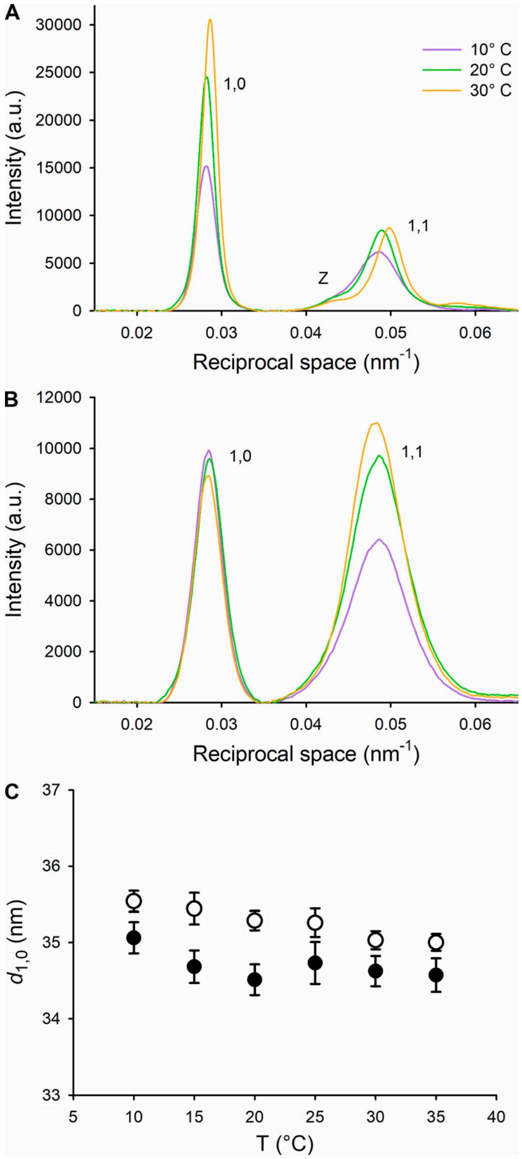

Figure S1. **Temperature dependence of the equatorial reflections. (A and B)** Equatorial intensity distributions from one EDL muscle at rest (A) and at the plateau of the isometric tetanus (B) at three different temperatures, as indicated by the color code in the inset. The main 1,0 and 1,1 reflections are indicated. At rest, the so-called Z-line reflection is also indicated (Z). Total exposure time, 40 ms at each temperature for both states. a.u., arbitrary units. **(C)** Temperature (T) dependence of the spacing of the 1,0 reflection, d_{10}, at rest (open circles) and at the plateau of the isometric tetanus (black circles). Mean ± SEM; six muscles.

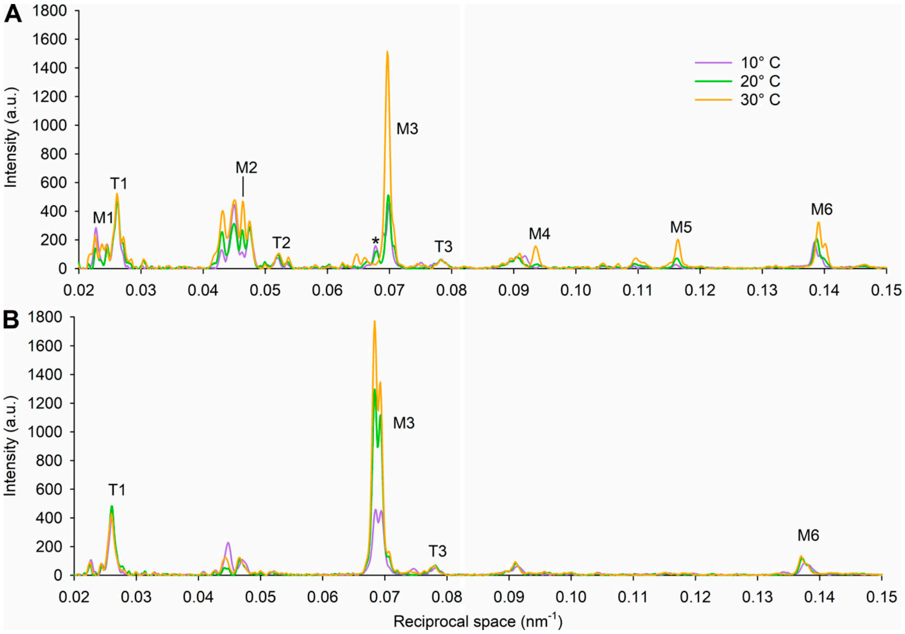

Figure S2. **Temperature dependence of the meridional reflections. (A and B)** Meridional intensity distributions from one EDL muscle, at rest (A) and at the plateau of the isometric tetanus (B) at three different temperatures, as indicated by the color code in the inset. The myosin-based (M) and troponin-based (T) reflections are indicated. *, A peak close to the M3 reflection but not part of it, present at rest at lower temperatures; a.u., arbitrary units.

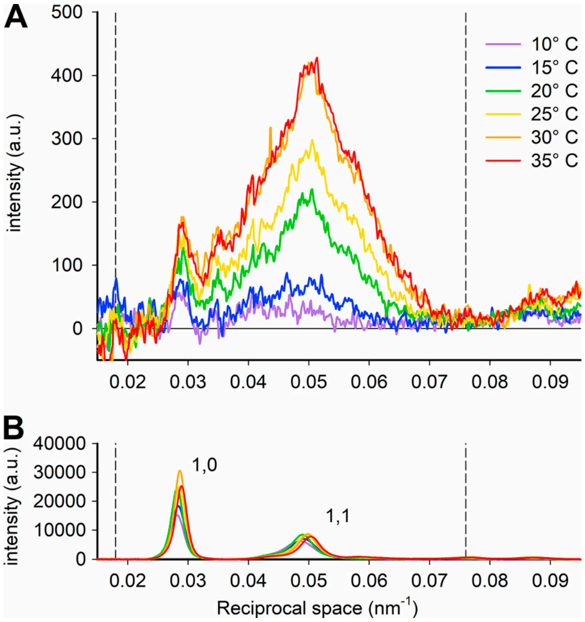

Figure S3. **Temperature dependence of the first-order myosin layer line at rest. (A)** Intensity distribution of the first-order myosin layer line (ML1) along the radial direction at rest at different temperatures as indicated by the color code in the inset. The intensity profiles were obtained by adding the patterns from six muscles (240-ms total exposure time for each intensity profile). Dashed vertical lines mark the integration limits used to obtain the intensity distribution in the direction parallel to the meridional axis. **(B)** Equatorial intensity distribution from one EDL muscle at rest (same as in Fig. S2) to show the sampling of the ML1 radial profile at the level of the 1,0 reflection. a.u., arbitrary units.

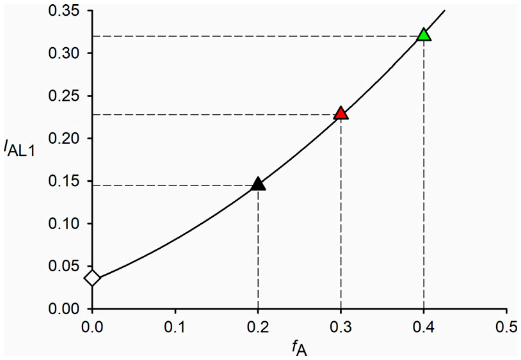

Figure S4. **Relation between the fraction of actin-attached motors, f_A, and the intensity of the first actin layer line, I_{AL1}, from the model of Koubassova et al. (2008).** I_{AL1} is normalized for the value in rigor condition, when all the motors are actin-attached (f_A = 1). The triangles show the model prediction for f_A = 0.4 (green), 0.3 (red), and 0.2 (black) at the plateau of an isometric tetanus (T_0). The diamond shows the model I_{AL1} for zero motors attached, corresponding to the resting state. I_{AL1} at rest relative to that at T_0 for f_A = 0.4, 0.3, and 0.2 is 11, 15, and 25%, respectively. Adapted from Fig. 6 A in Koubassova et al. (2008).

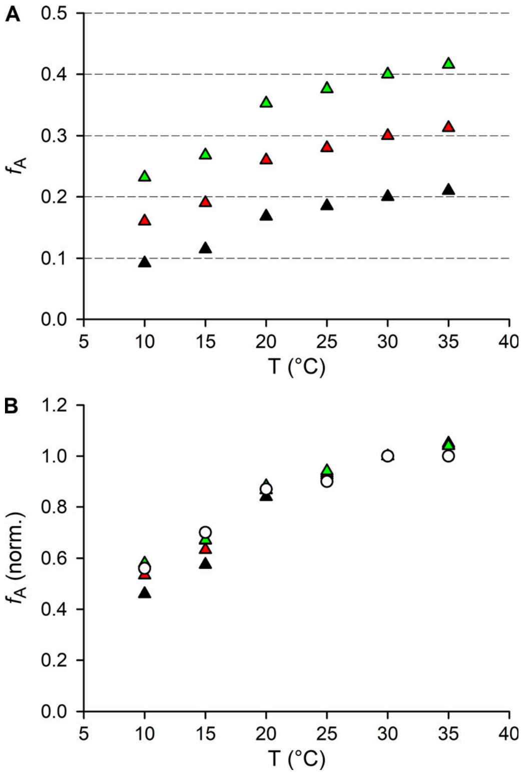

Figure S5. **Temperature dependence of the fraction of actin-attached motors (f_A, triangles) at the plateau of an isometric tetanus (T_0). (A)** The temperature (T) dependence of the observed intensity of the first actin-based layer line (I_{AL1}) was compared with the values calculated from the structural model of Koubassova et al. (2008). The colors indicate different assumptions for f_A at T_0 at 30°C ($f_{A,30}$): 0.2 black, 0.3 red, 0.4 green. **(B)** Triangles, the same data as in A, normalized for $f_{A,30}$ (same color code as in A). Circles, f_A calculated from the intensity of the M3 reflection (from Fig 6 B in Koubassova et al. [2008]).

References

Koubassova, N.A., S.Y. Bershitsky, M.A. Ferenczi, and A.K. Tsaturyan. 2008. Direct modeling of X-ray diffraction pattern from contracting skeletal muscle. *Biophys. J.* 95:2880–2894. https://doi.org/10.1529/biophysj.107.120832

Linari, M., M. Caremani, C. Piperio, P. Brandt, and V. Lombardi. 2007. Stiffness and fraction of Myosin motors responsible for active force in permeabilized muscle fibers from rabbit psoas. *Biophys. J.* 92:2476–2490. https://doi.org/10.1529/biophysj.106.099549
